# Alpha or beta human chorionic gonadotropin knockdown decrease BeWo cell fusion by down-regulating PKA and CREB activation

**DOI:** 10.1038/srep11210

**Published:** 2015-06-08

**Authors:** Sudha Saryu Malhotra, Pankaj Suman, Satish Kumar Gupta

**Affiliations:** 1 Reproductive Cell Biology Laboratory, National Institute of Immunology, New Delhi-110 067, India; 2 Amity Institute of Biotechnology, Amity University, Sector-125, Noida, Uttar Pradesh-201 301, India

## Abstract

The aim of the present study is to delineate the role of human chorionic gonadotropin (hCG) in trophoblast fusion. In this direction, using shRNA lentiviral particles, α- and β-hCG silenced ‘BeWo’ cell lines were generated. Treatment of both α- and β-hCG silenced BeWo cells with either forskolin or exogenous hCG showed a significant reduction in cell fusion as compared with control shRNA treated cells. Studies by qRT-PCR, Western blotting and immunofluorescence revealed down-regulation of fusion-associated proteins such as syncytin-1 and syndecan-1 in the α- and β-hCG silenced cells. Delineation of downstream signaling pathways revealed that phosphorylation of PKA and CREB were compromised in the silenced cells whereas, no significant changes in p38MAPK and ERK1/2 phosphorylation were observed. Moreover, β-catenin activation was unaffected by either α- or β-hCG silencing. Further, inhibition of PKA by H89 inhibitor led to a significant decrease in BeWo cell fusion but had no effect on β-catenin activation suggesting the absence of non-canonical β-catenin stabilization via PKA. Interestingly, canonical activation of β-catenin was associated with the up-regulation of Wnt 10b expression. In summary, this study establishes the significance of hCG in the fusion of trophoblastic BeWo cells, but there may be additional factors involved in this process.

Adequate maintenance of pregnancy is attributed to proper syncytial development through trophoblast cell fusion as it serves a crucial role in feto-maternal nutrient exchange and synthesis of steroid and peptide hormones like progesterone and human chorionic gonadotropin (hCG); essential for fetal growth and development^1^. This multinucleated layer is sustained throughout pregnancy by a continuous turnover of the underlying mononucleated cytotrophoblasts (CTB) which proliferate and fuse with the overlying syncytiotrophoblast (STB) with simultaneous apoptotic release as syncytial knots. Aberrations during syncytialization leads to several pregnancy related disorders such as preeclampsia and intrauterine growth restriction (IUGR)^2^,^3^. Various cytokines and growth factors regulate trophoblastic cell fusion either in an autocrine or paracrine manner^4^,^5^,^6^,^7^. Further, a few membrane proteins involved in direct cell to cell recognition and adhesion have been shown to play a role in syncytialization which include syncytin-1 and its receptors ASCT1 and ASCT2^8^,^9^, gap junction connexin 43^10^, CD98 and its receptor galectin 3^11^,^12^ and syndecan-1^13^.

After implantation, hCG is the first signal detected in the maternal blood and its expression increases progressively during the first trimester. Independent studies support its role in trophoblast fusion as exogenous addition of purified hCG to CTB isolated from term placentas led to increase in fusion; while concomitant addition of polyclonal antibodies against hCG suppressed fusion^14^,^15^. Similarly in trisomy 21 placentas, aberrant STB development was observed, which may be due to the presence of abnormal hCG and a decreased expression of luteinizing hormone/choriogonadotropin receptor (LHCGR)^16^,^17^. In general, hCG binds to LHCGR, a rhodopsin-like G protein-coupled receptor^18^ leading to an increase in cAMP via adenylyl cyclase^19^, which subsequently activates cAMP dependent PKA signaling. In trophoblastic cells, stimulation of PKA results in the up-regulation of glial cell missing a (GCMa) transcription factor which further activates syncytin-1 leading to cell fusion^20^. Apart from PKA, other signaling pathways are also known to be involved during syncytalization, like p38MAPK or MAPK11/14, ERK1/2 and Wnt/beta-catenin pathways^21^,^22^,^23^,^24^. Taking cue from all these independent studies, we wanted to investigate whether there is a differential expression in all or some of these pathways in those trophoblastic cells which inherently produce less hCG. This would reveal whether any cross communication among PKA/ p38MAPK/ ERK1/2/ β-catenin pathways exist or they function independently or may complement each other to achieve a common event of cellular fusion.

To achieve these goals, BeWo cells, an established *in vitro* model to study trophoblast fusion^25^,^26^ have been employed; using shRNA, α- and β-hCG-knockdown BeWo cell lines were generated. These cells were used to study the forskolin and hCG mediated cell fusion. Expression levels of different membrane proteins such as syncytin-1 and syndecan-1 that are responsible for cell fusion have been investigated by quantitative RT-PCR (qRT-PCR) and immunofluorescence/Western blotting. More so, differences in downstream signaling pathways between control and silenced cells were delineated to showcase critical molecules in hCG mediated cell fusion.

## Results

### Silencing of α- and β- subunits of hCG inhibits forskolin-mediated BeWo cell fusion

**Figure 1:**
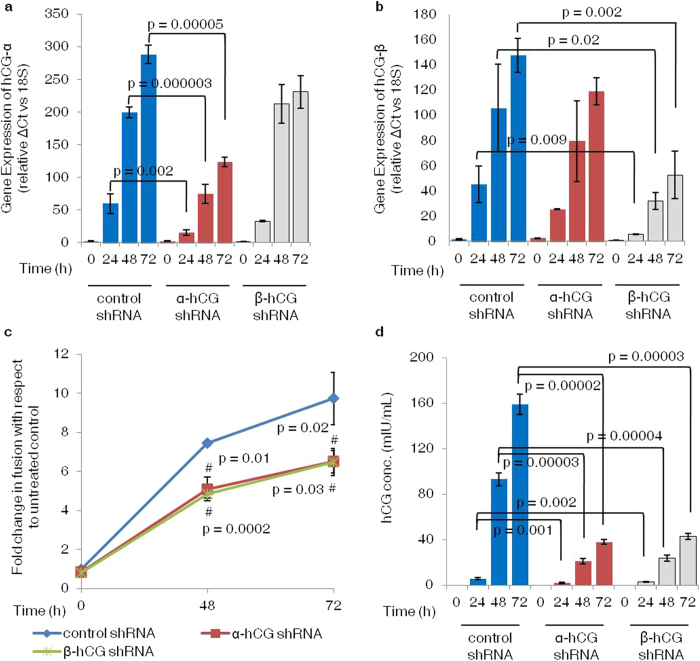
Effect of α- and β-hCG silencing on forskolin mediated syncytialization of BeWo cells. BeWo cells knockdown for α- and β-hCG were made using lentiviral shRNA as described in *Methods*. Efficacy of silencing of α- and β-hCG transcript was confirmed by qRT-PCR using specific primers and secreted hCG by ELISA. The effect of α- and β-hCG silencing on fusion was studied at 48 and 72 h of forskolin (25 μM) treatment by desmoplakin I + II staining. Panels (**a**) and (**b**) show qRT-PCR data comparing transcript levels of α- and β-hCG respectively, in control, α- and β-hCG silenced cells on foskolin treatment. Each bar represents relative ΔCt values after normalization with the 18 S rRNA, expressed as mean ± s.e.m. of three independent experiments performed in triplicates. Panel (**c**) compares the fold change in fusion on treatment with forskolin (25 μM) in control and α- and β-hCG silenced BeWo cells when compared with their respective untreated controls at 48 and 72 h. Values are shown as mean ± s.e.m. of three independent experiments. Panel (**d**) shows hCG secreted by control, α- and β-hCG silenced cells in response to forskolin treatment and represented as mean ± s.e.m. of three independent experiments performed in duplicates. ^#^p < 0.05 between control and silenced cells at respective time point.

To assess the importance of hCG in cell fusion, BeWo cell lines knocked-down for α- and β- subunits of hCG were developed using lentiviral shRNA transfection as described in *Methods*. These cells were treated with an optimized concentration of forskolin (25 μM) for 24, 48 and 72 h. Analysis of α-hCG silenced BeWo cells by qRT-PCR revealed a significant decrease in the levels of transcript for α-hCG. However, no significant effect on the β-hCG transcript levels was observed (Fig. 1a). Similarly, β-hCG silenced BeWo cells showed a significant decrease in the transcript for β-hCG without any significant changes in the α-hCG transcript (Fig. 1b). Western blot analysis of α-hCG silenced cells also confirmed decrease in the α-hCG without an excessive accumulation of β-hCG as compared to control shRNA cells. In the β-hCG silenced cells, a decrease in the β-hCG was also observed without any effect on the levels of α-hCG with respect to control (Supplementary Fig. S1 online). As compared to control shRNA transfected BeWo cells, both α- and β-hCG silenced BeWo cells showed a significant decrease in their fusion capacity as assessed by desmoplakin I + II staining both at 48 as well as 72 h after forskolin treatment (Fig. 1c). Representative desmoplakin I + II staining profiles of shRNA control, α- and β-hCG silenced cells at 0, 48 and 72 h with and without forskolin treatment is shown in Supplementary Fig. S2 online. Further, a concomitant decrease in the levels of secreted hCG by α- and β-hCG silenced cells was also observed (Fig. 1d). At 72 h after treatment with forskolin, in α- and β-hCG silenced cells, 38 ± 2.1 and 42.8 ± 2.8 mIU/mL of secreted hCG was observed respectively as compared to 159 ± 8.75 mIU/mL in the control shRNA treated BeWo cells. No statistically significant difference (p > 0.05) in the fusion efficiency and total hCG secretion between α- and β-hCG knockdown cells was observed in response to forskolin treatment.

### Expression profile of fusion associated proteins in α- and β-hCG silenced BeWo cells treated with forskolin

**Figure 2:**
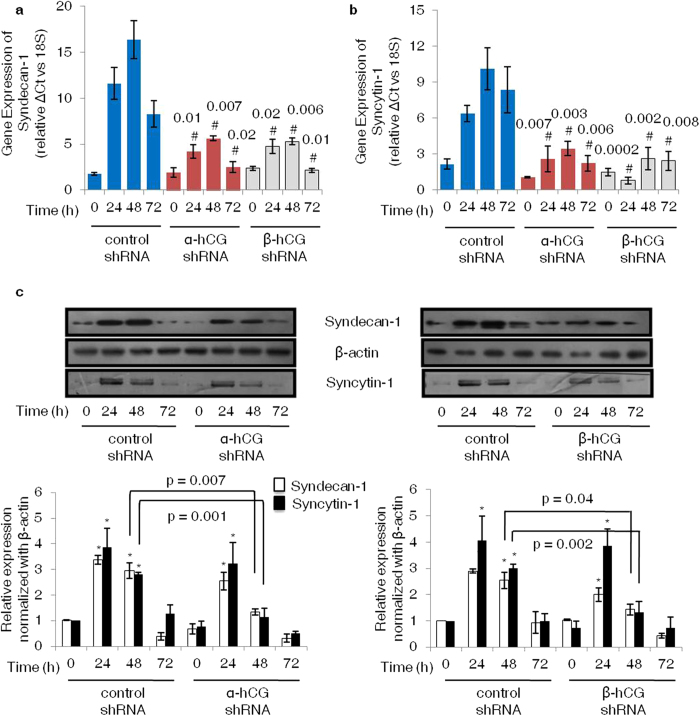
Effect of α- and β-hCG silencing on the expression of syncytin-1 and syndecan-1. Control, α- and β-hCG silenced cells were treated with forskolin (25 μM) for 24, 48 and 72 h. Subsequently, total RNA and cell lysates were prepared and profiles of syncytin-1 and syndecan-1 were determined by qRT-PCR and Western blotting. Panels (**a**) and (**b**) show qRT-PCR data comparing transcript levels of syncytin-1 and syndecan-1 respectively, in control, α- and β-hCG silenced cells after 24, 48 and 72 h of foskolin treatment. Each bar represents relative ΔCt values after normalization with the 18S rRNA, expressed as mean ± s.e.m. of three independent experiments performed in triplicates. Panel (**c**) represents comparative expression profiles of syncytin-1 and syndecan-1 between control and α- and β-hCG silenced cells on forskolin treatment with β-actin used as an internal control. The cropped blots were run under the same experimental conditions and the full length blots can be viewed in Supplementary Fig. S12 online. Values are expressed as mean ± s.e.m. of band intensity of three independent experiments. Representative blots for the same are appended with the graphs. ^#^p < 0.05 between control and silenced cells at respective time point; *p < 0.05 with respect to 0 h.

Syncytin-1 and syndecan-1 have been shown to play an important role in trophoblastic cell fusion^8^,^9^,^13^. We next examined, if silencing α- or β-hCG have also affected the expression levels of syndecan-1 and syncytin-1. Silencing of both α- and β-hCG led to a significant decrease in the transcripts of syndecan-1 and syncytin-1 at 24, 48 and 72 h of forskolin (25 μM) treatment as compared to control shRNA treated BeWo cells (Fig. 2a,b). Western blot analysis also confirmed that protein expression levels of both syncytin-1 and syndecan-1 were significantly lower at 48 h as compared to control cells (Fig. 2c). It was further reinforced by performing indirect immunofluorescence of these proteins in control and silenced cells which showed a similar expression profile as observed in Western blotting (Supplementary Fig. S3 online). Syndecan-1 was localized on the cell membrane whereas syncytin-1 was observed as intense punctate cytoplasmic and perinuclear deposits and in the cells undergoing fusion these were mainly localized on the cell surface.

In addition to syndecan-1 and syncytin-1, treatment of shRNA control, α- and β-hCG silenced BeWo cells with forskolin led to an increase in the expression of other fusion markers such as human placental lactogen (hPL) and cytochrome P-450 (CYP19A1) as revealed by qRT-PCR (Supplementary Fig. S4 online). Interestingly, the transcript for hPL was down-regulated in the α- and β-hCG silenced cells as compared to control cells. However, no significant difference in the levels of the transcript for CYP19A1 was observed among control and silenced cells treated with forskolin (Supplementary Fig. S4 online).

### Dose dependent increase in BeWo cell fusion by hCG and its effect on fusion efficiency of α- and β-hCG silenced cells

**Figure 3:**
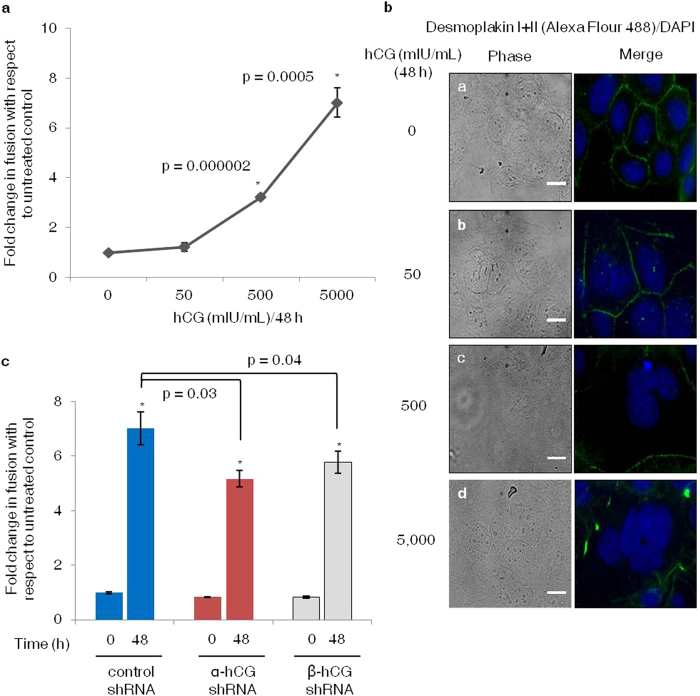
Induction of fusion by hCG in naïve BeWo cells as well as α- and β-hCG silenced cells. BeWo cells were treated with varying concentrations of hCG (0, 50, 500 and 5,000 mIU/mL) and percent fusion was estimated after 48 h by desmoplakin I + II staining as described in *Methods*. Panel (**a**) shows the effect of different concentrations of hCG on syncytialization of BeWo cells at 48 h. The data is expressed as mean ± s.e.m. of three independent experiments. Panel (**b**) represent images for the expression of desmoplakin I + II following 0, 50, 500, and 5,000 mIU/mL of hCG treatment as sub-panels a, b, c and d respectively. Panel (**c**) shows the effect of hCG (5,000 mIU/mL) treatment at 48 h of control shRNA as well as α- and β-hCG silenced BeWo cells on syncytialization. Data is expressed as mean ± s.e.m. of three independent experiments. Scale bar indicates 20 μm. *p < 0.05 with respect to 0 h.

Treatment of BeWo cells with varying concentrations of hCG (0, 50, 500 and 5,000 mIU/mL) for 48 h showed a dose dependent increase in BeWo cell fusion (Fig. 3a,b). Western blot analysis revealed that treatment of naïve BeWo cells with hCG (5 IU/mL) also led to a significant increase in the expression of fusion-associated markers such as syndecan-1 and syncytin-1 (Supplementary Fig. S5 online). In addition, treatment of naïve BeWo cells with hCG also led to an increase in expression of hPL and CYP19A1 (Supplementary Fig. S5 online). Further, fusion potential of α- and β-hCG silenced BeWo cells was assessed by exogenous addition of hCG (5 IU/mL). It was observed that although, the silenced cells showed an increase in fusion as compared to their respective untreated control at 48 h but it was significantly (p value < 0.05) less as compared with the control shRNA cells treated with hCG (Fig. 3c).

### Decrease in fusion efficiency of α- and β-hCG silenced BeWo cells may not be associated with their cell viability, proliferation and LHCGR expression

To further understand that the decrease in fusion efficiency of α- and β-hCG silenced cells in response to forskolin and exogenous hCG is not due to fundamental differences between the naïve and silenced cells, we performed viability assay by Sytox Red staining and proliferation assay using [^3^H]-thymidine for the naïve, control shRNA, α- and β-hCG silenced cells. We found no significant differences in the viability and proliferation of shRNA control cells, α-hCG silenced cells and β-hCG silenced cells as compared to the naïve BeWo cells (Supplementary Fig. S6 online). To further delineate, if differential expression of LHCGR in the silenced BeWo cells is responsible for reduction in their fusion efficiency, the transcript for LHCGR was determined by qRT-PCR. No significant difference in the LHCGR transcript was observed between control shRNA *versus* either α- or β-hCG silenced cells, suggesting that the reduction in fusion of the silenced cells may not be due to a decrease in the LHCGR expression (Supplementary Fig. S7 online).

### Silencing of α- and β-hCG does not alter phosphorylation profile of p38MAPK and ERK1/2 as compared to control shRNA treated BeWo cells

To see, if knockdown in the expression of α- or β-hCG in BeWo cells affects the signaling molecules P-p38MAPK (Thr180/Tyr182) and P-ERK1 (Thr202/Tyr204)/ P-ERK2 (Thr185/Tyr187) in response to forskolin, Western blots were carried out at 0, 0.5, 1 and 48 h. Expression of P-p38MAPK was significantly higher at 30 min in both α- and β-hCG silenced cells which was not significantly different than the control cells. Similarly, a significant increase in P-ERK1/2 was observed at 30 min and 1 h in both α- and β-hCG silenced BeWo cells, which was also observed in control cells (Supplementary Fig. S8 online). No significant difference in the activation of P-ERK1/2 was observed in the control *versus* the α- or β-hCG silenced BeWo cells.

### Silencing of α- and β-hCG led to reduction in the activated PKA and CREB upon forskolin treatment

**Figure 4:**
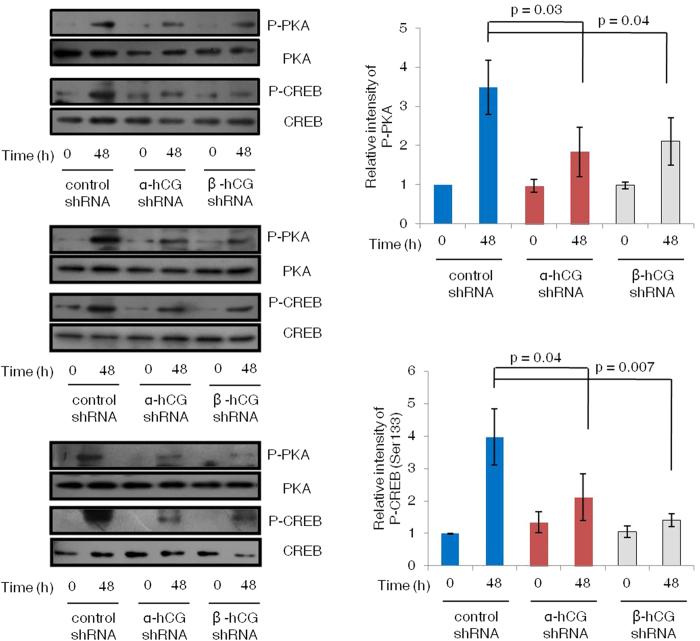
Expression profile of activated PKA and CREB in control and α- and β-hCG silenced BeWo cells treated with forskolin for 48 h. Control shRNA and α- and β-hCG silenced BeWo cells were treated with forskolin (25 μM) for 0 and 48 h and cell lysates were used to perform Western blots for phophorylated and non-phosphorylated forms of PKA and CREB. Blots from three independent experiments are shown. The cropped blots were run under the same experimental conditions and the full length blots can be viewed in Supplementary Fig. S13 online. Graphs represent the densitometric plots (mean ± s.e.m.) of P-PKA (Thr197) and P-CREB (Ser133). Band intensities were normalized with respect to their respective non-phosphorylated proteins and the data is expressed as fold change with respect to 0 h of control cells.

**Figure 5:**
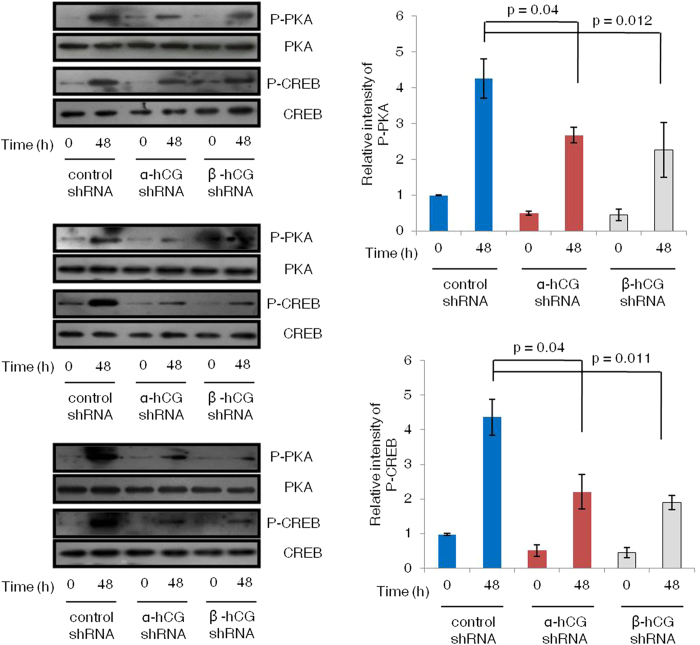
Signaling molecules in α- and β-hCG silenced BeWo cells treated with hCG. Control and α- and β-hCG silenced BeWo cells were treated with hCG (5 IU/mL) for 0 and 48 h and cell lysates were used to perform Western blots for phophorylated and non-phosphorylated forms of PKA and CREB. Blots from three independent experiments are shown. The cropped blots were run under the same experimental conditions and the full length blots can be viewed in Supplementary Fig. S14 online. Graphs represent the densitometric plots (mean ± s.e.m.) of P-PKA (Thr197) and P-CREB (Ser133). Band intensities were normalized with respect to their respective non-phosphorylated proteins and the data is expressed as fold change with respect to 0 h of control cells.

The α- and β-hCG silenced and control cells were treated with forskolin (25 μM) for varying time periods (0, 0.5, 1 and 48 h) and cell lysates were subjected to Western blotting. Expression of P-PKA (Thr197) and P-CREB (Ser133) in control cells increased significantly (p < 0.05) at all the time points subsequent to the treatment with forskolin that peaked at 30 min followed by a slight decrease at 1 and 48 h. Both α- and β-hCG silenced cells also showed a similar pattern at early time points (0.5 and 1 h) but, a significant reduction in its activation was observed at the later time interval (48 h) (Supplementary Fig. S9 online). The forskolin-mediated inhibition of P-PKA and P-CREB in α- and β-hCG silenced as compared to control shRNA BeWo cells at 48 h is shown in triplicate in Fig. 4. To strengthen our observation with forskolin in this model, we also used natural agonist (hCG) for these signaling pathways. On treatment with hCG (5 IU/mL), a significant decrease in the levels of both P-PKA and P-CREB at 48 h was also observed in the α- and β-hCG silenced cells as compared with the control shRNA BeWo cells (Fig. 5).

### Forskolin mediated up-regulation of β-catenin in BeWo cells may be associated with a concomitant increase in Wnt 10b, which is not affected by hCG subunit silencing

**Figure 6:**
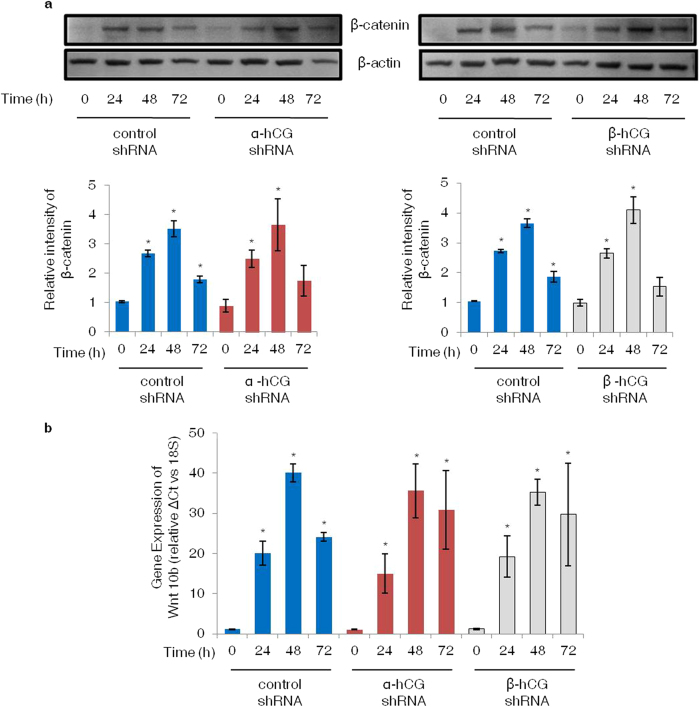
Expression profiles of β-catenin and transcript for Wnt 10b in α- and β-hCG silenced BeWo cells treated with forskolin. Control and α- and β-hCG silenced BeWo cells were treated with forskolin (25 μM) for 24, 48 and 72 h; cell lysates for Western blotting and total RNA for qRT-PCR were processed as described in *Methods*. Panel (a) shows densitometeric plots of β-catenin with β-actin used as an internal control. Data is represented as mean ± s.e.m. of at least three experiments. Representative blots for the same are also shown. Panel (b) represents Wnt 10b transcript profile at varying time points in the control and α- and β-hCG silenced BeWo cells. Each bar represents relative ΔCt values after normalization with the 18S rRNA, expressed as mean ± s.e.m. of three independent experiments performed in triplicates. *p < 0.05 with respect to 0 h

It is known that trophoblast fusion is associated with an increase in β-catenin via canonical Wnt signaling^24^. Cell lysates were prepared after 0, 24, 48 and 72 h of forskolin treatment and processed for Western Blotting. It led to a significant increase in β-catenin expression at 24, 48 and 72 h in the control shRNA cells. Moreover, β-catenin was also up-regulated in the forskolin treated α- and β-hCG silenced cells, which was not significantly different as compared to shRNA control cells at all the time points analyzed (Fig. 6a). Further, to check, which Wnt is up-regulated in BeWo cells treated with forskolin; total RNA was isolated from cells treated with forskolin at 0, 24, 48, and 72 h and qRT-PCR was performed for various Wnt ligands known to be expressed in the placenta. There was a significant increase in the expression of Wnt 10b in control as well as in α-/β-hCG knockdown cells to the same extent (Fig. 6b). Whereas, transcript levels of Wnt 3, 4, 5a, 7b and 11 decreased significantly within 24 h of forskolin treatment as compared to untreated control, in both control as well as α- and β- hCG silenced cells (Supplementary Fig. S10 online).

### Absence of non-canonical activation of β-catenin via PKA phosphorylation in forskolin mediated BeWo cells differentiation

**Figure 7:**
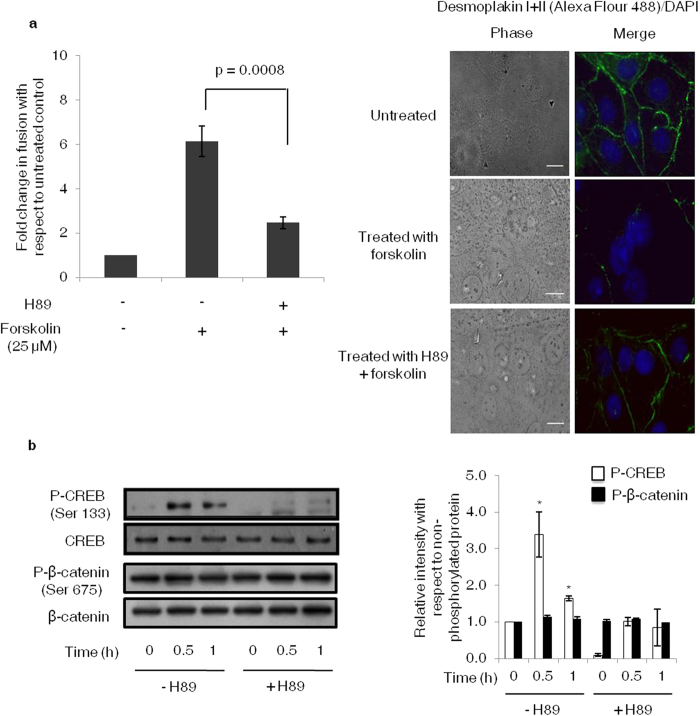
Effect of PKA inhibitor on BeWo cell fusion and expression profile of P-CREB and P-β-catenin (Ser675). BeWo cells were treated with H89 (10 μM) 1 h prior to forskolin (25 μM) treatment for varying time points using appropriate controls. Panel (**a**) represents fusion analysis carried out by desmoplakin staining at 48 h. Data is represented as fold change in fusion as compared with untreated control. Values are expressed as mean ± s.e.m. of three independent experiments. Representative desmoplakin I + II staining profile has also been shown. Scale bar represents 20 μm. Panel (**b**) shows representative Western blot and densitometric plot wherein band intensities normalized with their respective non-phosphorylated protein. The cropped blots were run under the same experimental conditions and the full length blots can be viewed in Supplementary Fig. S15 online. The data is expressed as fold change with respect to 0 h control and represents mean ± s.e.m. of three independent experiments. Representative blots for the same are also shown. *p < 0.05 with respect to 0 h.

To confirm if apart from canonical Wnt signaling, β-catenin stabilization in BeWo cells can be achieved by a cross-talk between PKA and β-catenin signaling, we inhibited PKA signaling by using H89 inhibitor which acts by blocking ATP site on the PKA catalytic subunit via competitive inhibition. A significant decrease in forskolin-mediated fusion of the cells treated with H89 was observed as compared with the untreated control (Fig. 7a). Activation of CREB was compromised in the H89 treated cells as early as 30 min. However, there was no change in the level of P-β-catenin (Ser675) between H89 treated and untreated cells (Fig. 7b). These observations suggest that non-canonical activation of β-catenin via PKA signaling is not involved in β-catenin stabilization during BeWo cell fusion.

## Discussion

The occurrence of clinical pathologies like preeclampsia, IUGR etc may result from defective syncytialization of CTBs because of imbalance in the expression of endometrial or trophoblast-derived growth factors and hormones at the site of implantation^2^,^3^. Expression of hCG starts during the course of cytotrophoblast differentiation^27^. From the time of conception an exponential increase in hCG levels in the maternal blood is observed, which increases from 5-50 mIU/mL in the first 7 days to 18-7340 mIU/mL at day 21. It is mainly produced by syncytiotrophoblast and a little by villous CTBs. Interestingly, hCG secreted by the STB acts in an autocrine manner to increase syncytium formation^14^,^15^. Receptors for hCG are also present on the endometrial cells and CTBs^28^ suggesting the possibility of its other diverse functions. In contrast to CTBs that undergo spontaneous *in vitro* fusion in medium containing fetal bovine serum, trophoblastic BeWo cells can be induced to differentiate^29^. cAMP activation via analogues of cAMP (dibutyryl cAMP) or inducers like forskolin, cholera toxin, Pertussis toxin, etc.^27^ are used to induce fusion *in vitro*. Fusion of BeWo cells was monitored by the loss of intercellular membrane desmoplakin staining as compared to its strong membrane and cytoplasmic staining in the mononucleated undifferentiated cells^30^. Forskolin-induced the fusion of BeWo cells as a function of time and this process was associated with concomitant increase in the level of hCG in the culture supernatant. This is indicative of the fact that cellular fusion and expression of hCG are interlinked and this has to get validated. To address the connection between expression of hCG and cellular fusion, we stably transfected BeWo cells with shRNA against α- as well as β- subunit of hCG separately. In this experiment, it was assumed that if any one of the subunit of hCG is silenced then, forskolin would not be able to induce the expression of functional hCG and cellular fusion should not get induced. Moreover, by silencing the two subunits individually, we also wanted to see differences (if any) in BeWo cell fusion. As per our assumption, forskolin-mediated experimental induction of fusion was significantly less in BeWo cells silenced for the expression of either α- or β- subunit of hCG to a similar extent as compared to the control (Fig. 1c). Further, expression of hCG in α-/β-hCG silenced cells was also significantly less as compared to the control (Fig. 1d).

These observations thus prompted us to hypothesize that forskolin-mediated signaling process induces the expression of hCG that amplifies the cellular fusion. In such a case, blocking of LHCGR by bio-neutralizing antibodies will attenuate the forskolin mediated BeWo cell fusion. Indeed, pre-incubation of naïve BeWo cells with bio-neutralizing rabbit polyclonal antibodies raised against Leucine Rich Repeats 1-6 of the human LHCGR (kindly made available by Prof. Rajan Dighe, Indian Institute of Science, Bangalore, India), significantly reduced the forskolin mediated cell fusion (Supplementary Fig. S11 online). These results suggest that during differentiation of BeWo cells as the hCG production increases, it also acts in an autocrine manner and helps in the fusion of BeWo cells as observed for STB^14^,^15^.

Cellular communications through cell to cell contact by the expression of several membrane proteins is the key to trophoblastic syncytialization. Syncytin-1 is an endogenous retroviral gene specifically expressed in the placenta and codes for a transmembrane protein found to be critical for trophoblast fusion as antibodies against it or its antisense oligonucleotides are known to abrogate fusion^8^. Also preeclamptic placental villi and trisomy 21 placentas show a reduced expression of syncytin-1^2^,^17^. Similarly, syndecan-1 a membrane bound proteoglycan associated with cytoskeleton remodeling and cell adhesion is known to be involved in BeWo cell fusion as its silencing by antisense oligonucleotides lead to a reduced fusion^13^. Moreover, 47% of the placentas from pre-eclamptic women showed very faint or undetectable syndecan-1 expression, in contrast to the normal placentas where 93% exhibited strong immunoreactivity for the syndecan-1^31^. In the current study, forskolin induced the expression of proven fusogenic membrane proteins like syncytin-1 and syndecan-1. In addition, forskolin treatment also leads to an increase in the expression of hPL and CYP19A1. However, in the cells silenced for α- and β-hCG, forskolin-mediated expression of syncytin-1, syndecan-1 and hPL was significantly less as compared to the control cells suggesting their relevance in cell fusion downstream of hCG. It is likely that CYP19A1 expression may not be regulated by hCG as both α- as well as β-hCG silenced BeWo cells did not show a significant reduction in its expression on treatment with forskolin.

The above observations suggested that the cells in which hCG production was compromised, were also expressing lower concentration of the fusogenic-proteins (except CYP19A1). Further, to reinforce the role of hCG in cell fusion, we questioned, whether exogenous supplementation of hCG to the α-/β-hCG silenced cells will rescue their fusion potential? hCG concentration that could induce the cellular fusion was optimized by analyzing the cellular fusion at different concentrations of hCG. As observed with forskolin, hCG also induced a dose dependent increase in cell fusion (Fig. 3a) and it was maximum with 5 IU concentration and that was selected for further experiments. Treatment of naïve BeWo cells with hCG also led to an increase in the fusion associated markers such as syndecan-1, syncytin-1, hPL and CYP19A1 (Supplementary Fig. S5 online). Exogenous supplementation of hCG in the α-/β-hCG silenced BeWo cells, though induced cellular fusion but not to the same extent as observed in control shRNA transfected cells (Fig. 3c). These observations prompted us to investigate the signaling intermediates that play crucial role in the forskolin/hCG mediated expression of fusogenic proteins and cell fusion.

Syncytialization is regulated by various cytokines, growth factors and hormones through modulation of various signaling pathways. ERK1/2 and p38MAPK have been suggested to play significant role in initiating trophoblast differentiation. Specific inhibitors for ERK1/2 and p38MAPK impaired syncytialization in primary trophoblast cultures^32^. Interestingly, in response to forskolin (25 μM) treatment, activation profile of p38 MAPK and ERK1/2 signaling molecules was found to be similar in case of both control and α-/β-hCG knockdown BeWo cells (Supplementary Fig. S8 online). These results suggest that activation of ERK1/2 and p38MAPK though involved in fusion of trophoblastic cells but, may not be critical for hCG mediated cell fusion. However, a significant reduction was observed in the activation of P-PKA and P-CREB in the α- and β-hCG silenced BeWo cells as compared to the control shRNA treated cells on treatment with either forskolin or exogenous hCG (Figs. 4,5; Supplementary Fig. S9 online). The reduction in the fusion efficiency of α- and β-hCG silenced BeWo cells treated with either forskolin or hCG may not be due to fundamental differences in the silenced cells as compared to either naïve or control shRNA treated BeWo cells as no significant difference in their cell viability, proliferation capability or expression of LHCGR was observed. These observations suggested that activation of PKA and CREB are of utmost importance in hCG mediated BeWo cell fusion. But, do other signaling work in tandem during hCG mediated fusion event?

β-catenin signaling has also been associated with trophoblast fusion as it directly targets the GCMa/syncytin pathway. Additionally, syncytin-1 is significantly down-regulated in the placenta of BCL9L-deficient mice where trophoblast fusion was highly compromised^24^. Moreover, in trophoblastic tissues from preeclamptic patients, β-catenin expression was found to be down-regulated whereas, dickkopf-related protein 1 and secreted frizzled-related protein were found to be elevated^33^,^34^. In an attempt to see whether silencing of α-/β- subunits of hCG had an impact on β-catenin expression in response to forskolin; we observed that though β-catenin expression increased significantly at 24 and 48 h but, there was no difference between the control and the silenced cells (Fig. 6a). This suggest that forskolin mediated expression of β-catenin has no bearing with the hCG mediated cell fusion. It can also be hypothesized that up-regualtion of β-catenin is an outcome of the forskolin induced expression of some of the Wnt isoform in BeWo cells. Wnt ligands are a family of hydrophobic cysteine rich secreted proteins, having a critical role in cell proliferation, cell death and differentiation. Fourteen out of 19 Wnt ligands have been detected in first trimester placental tissue^35^, showing a varied expression pattern between different trophoblast lineages. So far, it is known that canonical Wnt signaling is involved in trophoblast fusion but, association with specific Wnt ligand(s) was not well established. Therefore, we screened for the expression of various placenta specific Wnt ligands by qRT-PCR and found that Wnt 10b increased significantly at 24, 48 and 72 h upon forskolin treatment (Fig. 6b) irrespective of hCG silencing.

Earlier studies suggested the possibility of non-canonical activation and stabilization of β-catenin by cAMP/PKA dependent phosphorylation at serine 675; which led to inhibition of β-catenin ubiquitination and degradation and hence increasing its nuclear availability which further led to β-catenin target activation^36^,^37^,^38^. To explore such possibility in case of BeWo cells, we used H89 PKA inhibitor. H89 remarkably reduced the phosphorylation of CREB leaving the β-catenin (Ser675) phosphorylation unaffected in both the H89 treated and untreated cells confirming the absence of non-canonical activation of β-catenin via PKA signaling.

**Figure 8:**
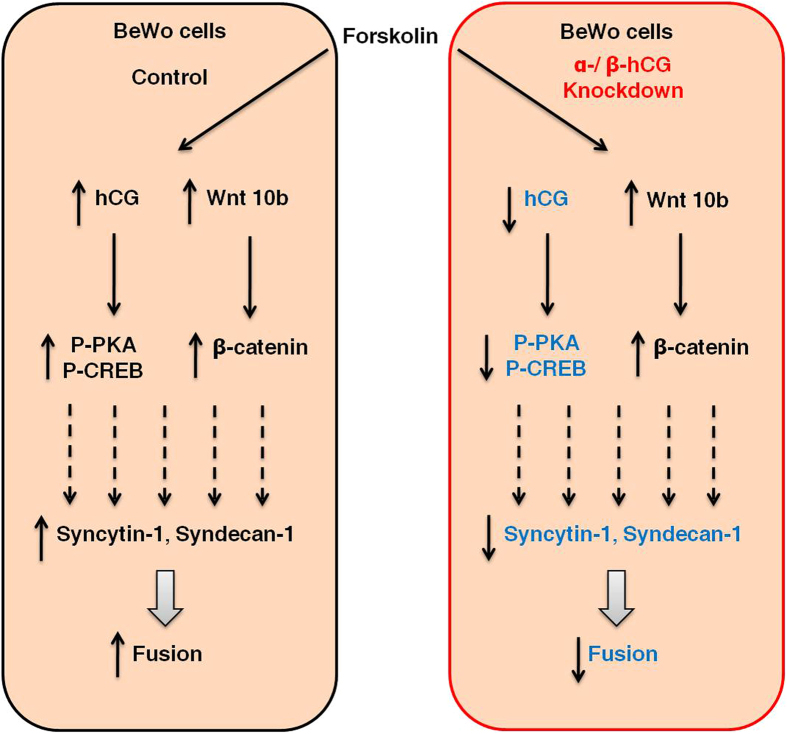
Schematic representation highlighting the differences and commonalities during fusion between control and α-/β-hCG knockdown BeWo cells. Treatment of BeWo cells with forskolin leads to an increase in fusion in the control shRNA transfected cells whereas, fusion is decreased in cells where α-/β- subunits of hCG were silenced. Expression of fusion potentiating proteins like syncytin-1 and syndecan-1 are markedly reduced in the α-/β-hCG silenced BeWo cells. In the α-/β-hCG silenced cells, as a result of reduced hCG secretion, activation of PKA and CREB signaling molecules is compromised. There is an increase in β-catenin expression in both control and hCG silenced cells alike, which can be associated with increase in transcript levels of Wnt 10b to a similar extent suggesting Wnt/β-catenin signaling is independent of hCG in case of BeWo cell fusion. Activation of p38 MAPK and ERK1/2 is also not critical for hCG-mediated fusion of BeWo cells (not shown in the schematic diagram).

To conclude, our study emphasizes the importance of both α- and β-hCG in BeWo cell fusion as down-regulation of either hampers fusion, which is marked by a decrease in syncytin-1, syndecan-1 and hPL expression. However, hCG may not be the only factor responsible for BeWo cell fusion. Our studies also showed that activation of PKA/CREB are critical for hCG mediated BeWo cell fusion and activation of ERK1/2 or p38MAPK or β-catenin signaling pathways may be playing supporting role during fusion. The relevance of canonical activation of β-catenin by Wnt 10b ligand needs further validation by silencing Wnt 10b and studying its impact on cell fusion. An overview of the forskolin/hCG mediated activation of downstream signaling, gene expression and outcome in terms of cell fusion is schematically presented in the Fig. 8 which summarizes that forskolin/hCG-PKA-CREB-syndecan-1/syncytin-1 axis is crucial for the fusion of trophoblastic BeWo cells.

## Methods

### Culture of BeWo cells

BeWo cells (American Type Culture Collection; ATCC, VA, USA) were cultured in Ham’s F-12 (Sigma-Aldrich Inc., St. Louis, MO, USA) medium supplemented with the antibiotic antimycotic cocktail (100 U⁄mL Penicillin; 100 μg⁄mL streptomycin; 0.25 μg⁄mL amphotericin B; Biological Industries, Kibbutz beit Haemek, Israel) and 10% heat-inactivated FBS (Gibco®, Life Technologies, CA, USA) under humidified conditions of 5% CO_2_ in air at 37 °C. Cells (~0.5–1.0 × 10^5^) were seeded into 25 cm^2^ culture flask (Greiner Bio-one GmbH, Frickenhausen, Germany) and passaged at 70–80% confluency.

### Gene silencing by shRNA

BeWo cells (0.5 × 10^4^/well) were seeded 24 h prior to transfection in 96-well plate in Ham’s F-12 medium with 10% FBS. Cells were transfected with α- and β-hCG shRNA or scrambled shRNA Lentiviral Particles (Santa Cruz Biotechnology Inc., Dallas, Texas, USA) at multiplicity of infection (MOI) of 5 using procedure as described by the manufacturer. Stables clones expressing shRNA were selected by growing in the presence of optimized concentration of puromycin dihydrochloride (0.5 μg/mL; Sigma-Aldrich Inc.).

### ***In vitro*** differentiation of BeWo cells

Cells (0.3 × 10^5^⁄well) were grown on cover slips in 24-well culture plates in Ham’s F-12 medium supplemented with 10% FBS. After 24 h, cells were washed and starved for 4 h with Ham’s F-12 medium without FBS. For induction of differentiation, cells were further cultured in serum-free Ham’s F-12 medium supplemented with 1 × ITS + 1 (Sigma-Aldrich Inc.) containing insulin (10 μg⁄mL), transferrin (5.5 μg⁄mL), selenium (0.005 μg⁄mL), linoleic acid (4.7 μg⁄mL), and BSA (500 μg⁄mL) with an optimized concentration of forskolin (25 μM; Sigma-Aldrich Inc.) or varying doses of hCG (Sigma-Aldrich Inc.) for 48 and 72 h keeping appropriate vehicle control. After each time point, conditioned medium was harvested and the cells were fixed with chilled methanol for 5 min at 4 °C and processed for desmoplakin I + II staining as described previously^13^. Slides were screened for immunofluorescence under a fluorescent phase contrast microscope (Nikon Instruments Inc., Melville, NY, USA) and images were captured using the Image Proplus software (Nikon). For each experimental group, fused cells were counted from 8 to 10 randomly selected microscopic fields. For each microscopic field, number of nuclei in the syncytium and total number of nuclei in that microscopic field were counted. From this, the percentage of fused cells was calculated by taking a ratio of the total number of nuclei in syncytium to the total number of nuclei in that microscopic field followed by multiplication with 100.

### Quantification of hCG in the conditioned medium

The harvested conditioned medium as described above was centrifuged at 3000 × g for 5 min to remove cell debris and stored at −20 °C until use. Solid phase sandwich ELISA was performed for the estimation of hCG level using DRG β-hCG ELISA kit following manufacturers’ instructions (DRG Instruments GmbH, Marburg, Germany).

### Quantitative real-time reverse transcription-polymerase chain reaction

BeWo cells (0.1 × 10^6^/well) were seeded in 6-well culture plates and cultured for 24 h. Cells were serum starved for 4 h before addition of forskolin (25 μM; Sigma-Aldrich Inc.) in Ham’s F-12 medium supplemented with 1 × ITS + 1 (Sigma-Aldrich Inc.) for 24, 48 and 72 h, keeping appropriate vehicle control. Total RNA was isolated from cells using Tri reagent (Sigma-Aldrich Inc.) following the standard protocol employing chloroform-isopropanol-ethanol steps for purification. Isolated RNA samples were quantitated by NanoDrop 3300 spectrophotometer (Thermo Scientific, NanoDrop Products, Wilmington, DE, USA) and were subjected to DNase I (Fermentas, Burlington, Ontario, Canada) treatment at 37 °C for 15 min as per the manufacturer’s instruction. The isolated RNA (5 μg) was used to prepare complementary DNA using a mix of random hexamers and oligo (dT) 18 primers, dNTP mixture, RT buffer and Maxima reverse transcriptase following the manufacturer’s protocol (Fermentas). The qRT-PCR reactions for analysis of transcripts for α-hCG, β-hCG, syncytin-1, syndecan-1, various wnt ligands, hPL, CYP19A1 and LHCGR were carried out in triplicates in 20 μl reaction mixture containing Maxima™ SYBR green qPCR master mix (Fermentas), synthesized complementary DNA and gene specific primers (1 nM) on Stratagene Mx3005P (Agilent Technologies Inc., Santa Clara, CA, USA ). The primers used for qRT-PCR and their respective annealing temperatures are listed in Supplementary Table S1 online. The temperature profiles used for the amplification of target sequences were: initial denaturation for 95 °C for 10 min, followed by 40 cycles of 95 °C for 15 sec, amplification for 1 min at primer specific annealing temperature value (Supplementary Table S1 online) and then a final melting curve analysis with a range from 60 to 95 °C over 20 min. Gene-specific amplification was confirmed by a single peak in the dissociation curve. Average threshold cycle (Ct) values for 18S rRNA (run in parallel reactions to the genes of interest) were used to normalize average Ct values of the gene of interest. These values were used to calculate the average for each group, and the relative ΔCt was used to determine the change in expression between the groups.

### Western blotting

Cells (0.1 × 10^6^/well) were cultured in 6-well culture plates for 24 h and starved of FBS for at least 4 h before treatment with forkolin (25 μM; Sigma-Aldrich Inc.) for varying time points in Ham’s F-12 medium supplemented with 1 × ITS + 1 (Sigma-Aldrich Inc.). After each time point, the medium was removed and cells were lysed in 100 μl of lysis buffer (20 mM Tris-HCl, 10% glycerol, 0.2 mM EDTA, 0.137 M NaCl, 1% NP-40) supplemented with complete protease and phosphatase inhibitor cocktail (Roche Diagnostics GmbH, Mannheim, Germany). This was followed by 3 rapid freeze and thaw cycles to ensure the complete cell lysis. Cell lysates were centrifuged at 12,000 × g for 10 min at 4 °C and the supernatant was collected. The amount of protein in each sample was quantitated by BCA colorimetric assay using BSA as standard (Thermo Fisher Scientific, Waltham, MA, USA). Cell lysates (40 μg/lane) were resolved by 0.1% SDS-12% polyacrylamide gel electrophoresis (SDS–PAGE) and processed for Western blotting as described previously^39^. After transfer of proteins, the nitrocellulose membrane was blocked in 5% BSA in TBST (50 mM Tris-HCl, 150 mM NaCl, 0.1% Tween-20; pH-7.4) and further incubated at 4 °C overnight with an optimized dilution of 1:1000 of rabbit polyclonal antibodies against phospho-PKA (P-PKA (Thr197)), PKA, P-CREB (Ser133), CREB, P-p38MAPK (Thr180/Tyr182), p38MAPK, P-ERK1 (Thr202/Tyr204)/ P-ERK2 (Thr185/Tyr187), ERK1/2, P-β-catenin (Ser675), β-catenin and mouse monoclonal antibody against β-actin (All from Cell Signaling Technology Inc., Danvers, MA, USA) in TBST containing 5% BSA. After subsequent washings with TBST, membrane was further incubated with 1:2000 dilution of HRP-conjugated anti-rabbit/mouse IgG antibody (Thermo Fisher Scientific) in TBST containing 5% BSA for 1 h at room temperature.

In another set of experiment, blots were also probed with rabbit polyclonal antibody against syndecan-1 (1 μg/mL; Invitrogen Corp., Carlsbad, CA, USA) and syncytin-1 (1:500 dilution; Thermo Fisher Scientific). These blots were further re-probed with mouse monoclonal antibody against β-actin (Cell Signaling Technology Inc.) at a dilution of 1:1000 followed by washing and incubation with 1:2000 dilution of HRP-conjugated anti-mouse IgG antibody (Thermo Fisher Scientific). In addition, blots were also probed with mouse monoclonal antibodies (10 μg/mL) against α-hCG^40^ and β-hCG^41^ and processed as described above. Blots were developed using chemiluminescent substrate: Immobilon (Millipore Corp., Billerica, MA, USA) and Hyperfilm-MP (GE Healthcare Bio-Sciences Pittsburg, PA, USA) as per the manufacturer instructions or using 0.05% 3-3’-diaminobenzidine (DAB) substrate (Sigma-Aldrich Inc.) in 50 mM PBS containing 0.1% hydrogen peroxide. Intensity of bands on Western blots was quantified by ImageJ software (http://rsb.info. nih.gov/ij/). Blots from at least three independent experiments were analyzed by normalizing the phosphorylated proteins with their non-phosphorylated counterparts and for other proteins with β-actin. Thereafter, fold change for all values including 0 h of the silenced cells was determined with respect to 0 h of the control shRNA treated cells.

### H89 mediated inhibition of PKA signaling

BeWo cells (0.3 × 10^5^/well; on cover slip in a 24-well plate for desmoplakin I + II staining or 0.1 × 10^6^/well; in a 6-well plate for Western blotting) were seeded overnight. Further, the cells were starved of FBS for at least 4 h before treatment with a pharmacological inhibitor of PKA, H89 dihydrochloride (10 μM; Sigma-Aldrich Inc.) for 1 h at 37 °C with 5% CO_2_ in humidified air. Subsequently, cells were washed and Ham’s F-12 medium supplemented with 1 × ITS + 1 (Sigma-Aldrich Inc.) and forskolin (25 μM; Sigma-Aldrich Inc.) was added for varying time points. Cells on coverslips were fixed in chilled methanol for 5 min at 4 °C for desmoplakin I + II staining or cell lysates were prepared for Western blotting. Appropriate controls were processed simultaneously.

### Statistical Analysis

Statistical analyses were performed using one-way ANOVA, and P < 0.05 was considered statistically significant. Values are given as mean ± standard error of the mean (s.e.m.) of at least three different experiments.

## Additional Information

**How to cite this article**: Saryu Malhotra, S. *et al.* Alpha or beta human chorionic gonadotropin knockdown decrease BeWo cell fusion by down-regulating PKA and CREB activation. *Sci. Rep.* **5**, 11210; doi: 10.1038/srep11210 (2015).

## Supplementary Material

Supplementary Information
