# Supplementary material for: Alpha or beta human chorionic gonadotropin knockdown decrease BeWo cell fusion by down-regulating PKA and CREB activation
Source: Sci Rep. 2015 Jun 8;5:11210. doi: 10.1038/srep11210 (PMC4459146; doi:10.1038/srep11210)
Supplement: Supplementary Information [file srep11210-s1.pdf]

Alpha or beta human chorionic gonadotropin knockdown decrease BeWo cell fusion by down-regulating PKA and CREB activation

Sudha Saryu Malhotra[1], Pankaj Suman[2] and Satish Kumar Gupta[1]*

[1]Reproductive Cell Biology Laboratory, National Institute of Immunology, New Delhi-110 067, India

[2]Amity Institute of Biotechnology, Amity University, Sector-125, Noida, Uttar Pradesh-201 301, India

*Corresponding Author

Correspondence should be addressed to Dr. S.K. Gupta. Email: skgupta@nii.ac.in

Reproductive Cell Biology Laboratory, National Institute of Immunology, Aruna Asaf Ali Marg, New Delhi-110 067, India

Tel: +91 11 26741249

Fax: +91 11 26742125

Figure S1

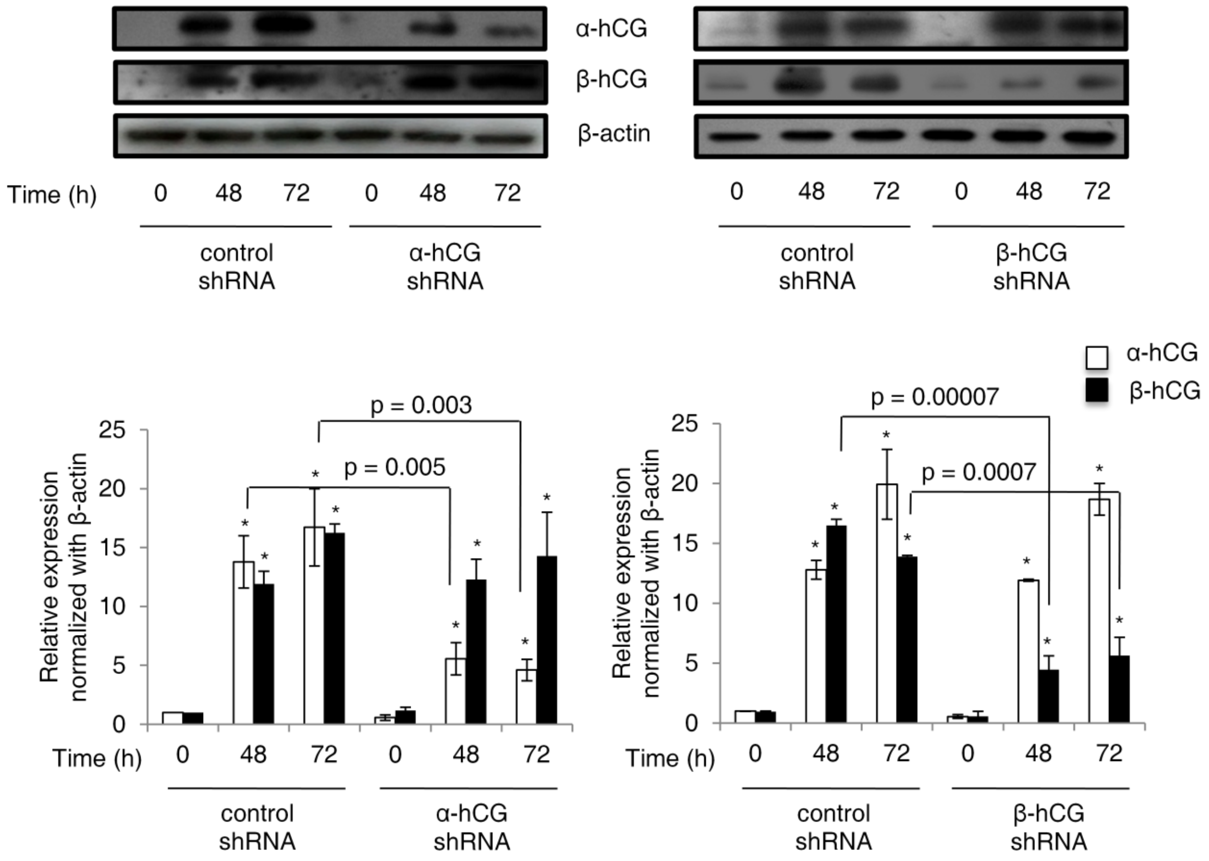
α-hCG
β-hCG
β-actin
Time (h)
0
48
72
0
48
72
control shRNA
α-hCG shRNA
0
48
72
0
48
72
control shRNA
β-hCG shRNA
α-hCG
β-hCG
p = 0.003
p = 0.005
Relative expression normalized with β-actin
25
20
15
10
5
0
Time (h)
0
48
72
0
48
72
control shRNA
α-hCG shRNA
p = 0.00007
p = 0.0007
Relative expression normalized with β-actin
25
20
15
10
5
0
Time (h)
0
48
72
0
48
72
control shRNA
β-hCG shRNA

Figure S2

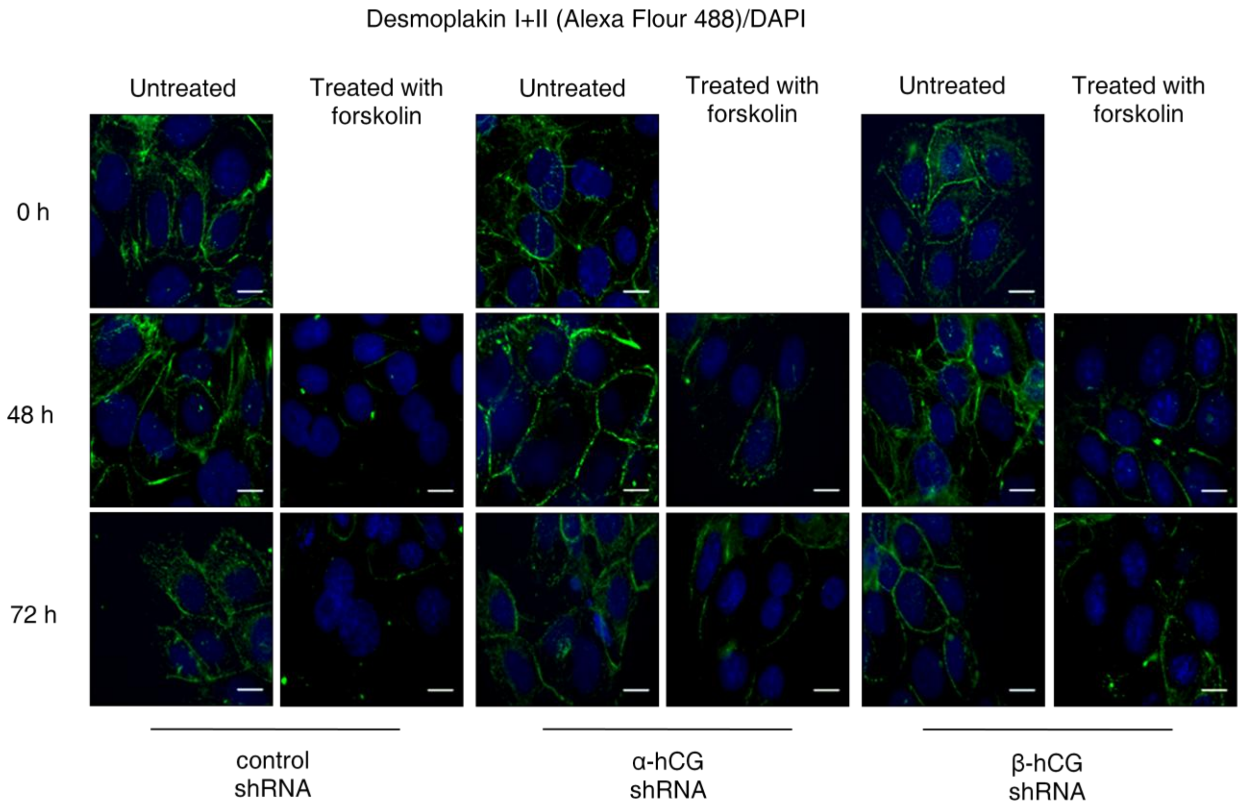
Desmoplakin I+II (Alexa Flour 488)/DAPI
Untreated
Treated with forskolin
Untreated
Treated with forskolin
Untreated
Treated with forskolin
0 h
48 h
72 h
control shRNA
α-hCG shRNA
β-hCG shRNA

Figure S3

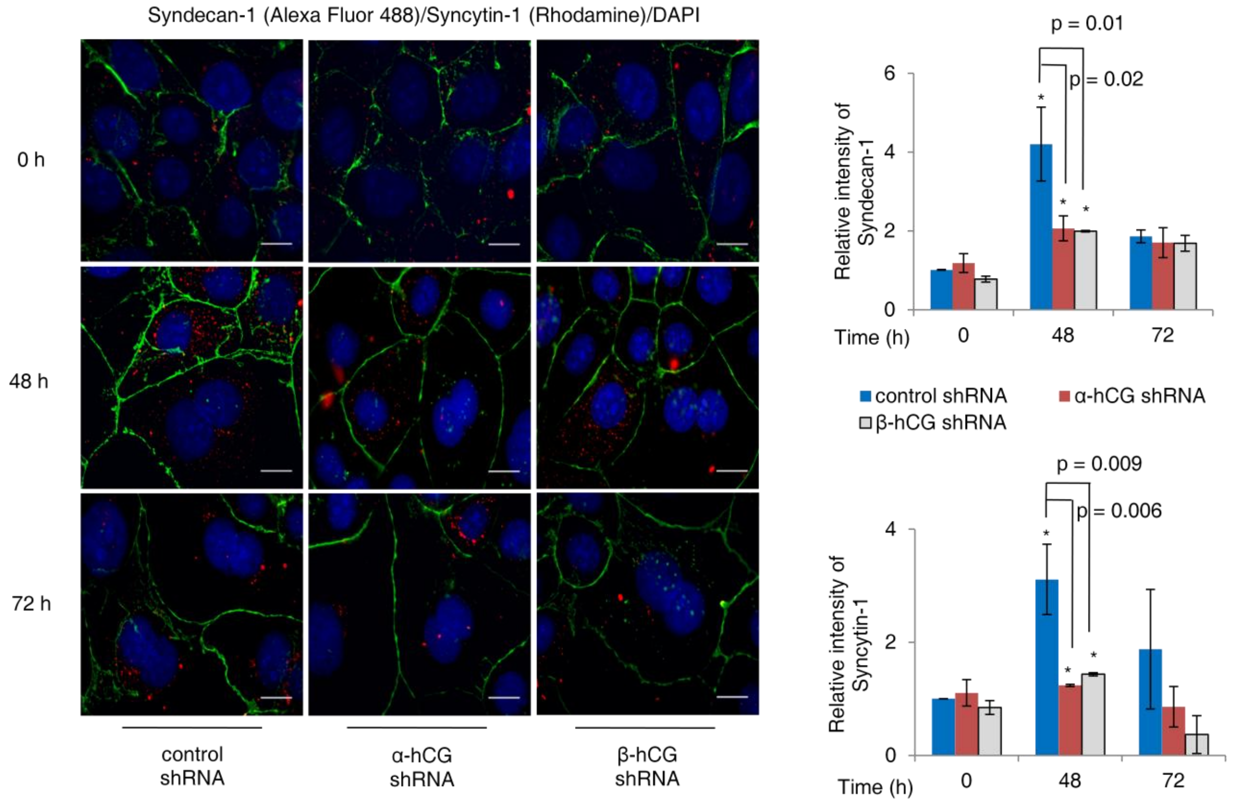

Figure S4

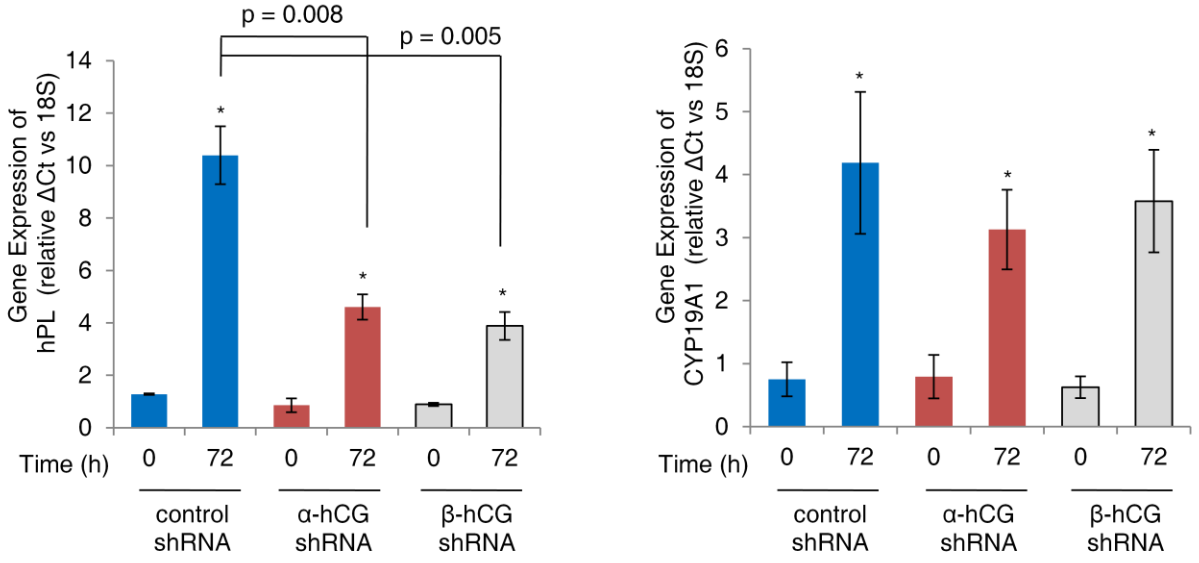

Figure S5

a

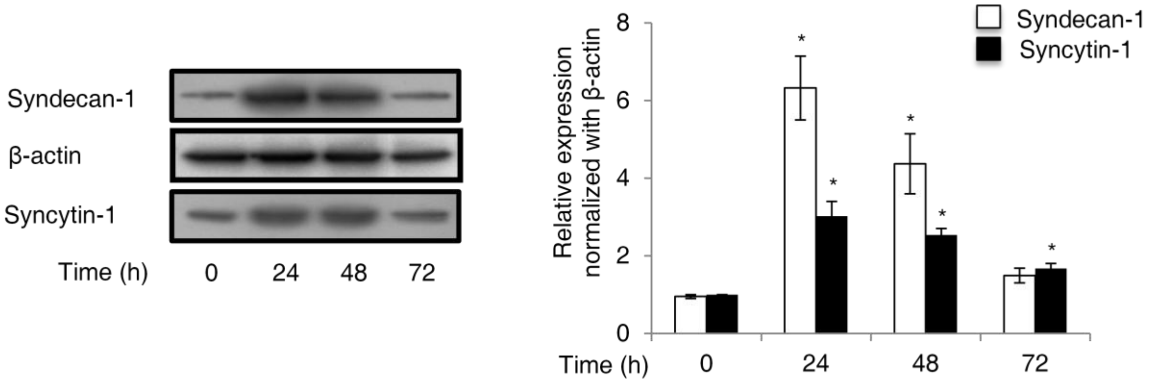
Syndecan-1
β-actin
Syncytin-1
Time (h)
0
24
48
72
Relative expression normalized with β-actin
8
6
4
2
0
Syndecan-1
Syncytin-1
Time (h)
0
24
48
72

b

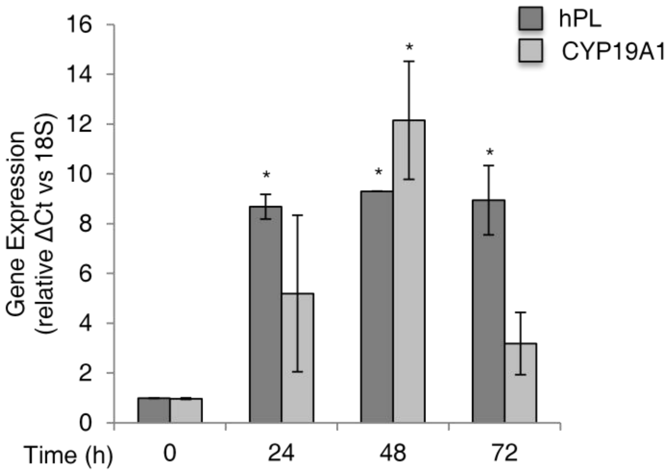
hPL
CYP19A1
Gene Expression (relative ΔCt vs 18S)
16
14
12
10
8
6
4
2
0
Time (h)
0
24
48
72

Figure S6

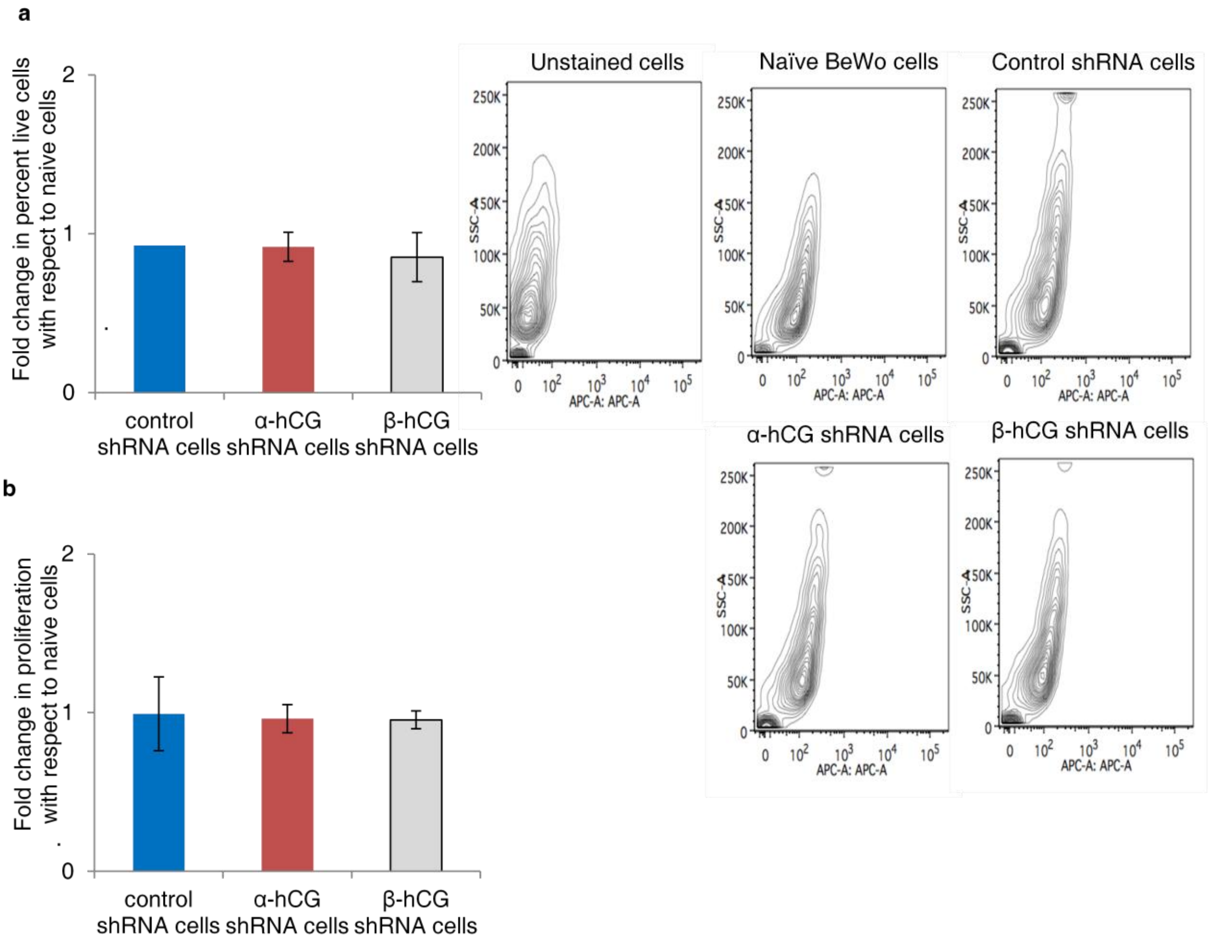
a
Fold change in percent live cells with respect to naive cells
2
1
0
control shRNA cells
α-hCG shRNA cells
β-hCG shRNA cells
Unstained cells
Naïve BeWo cells
Control shRNA cells
α-hCG shRNA cells
β-hCG shRNA cells
SSC-A
APC-A: APC-A
250K
200K
150K
100K
50K
0
b
Fold change in proliferation with respect to naive cells
2
1
0
control shRNA cells
α-hCG shRNA cells
β-hCG shRNA cells

Figure S7

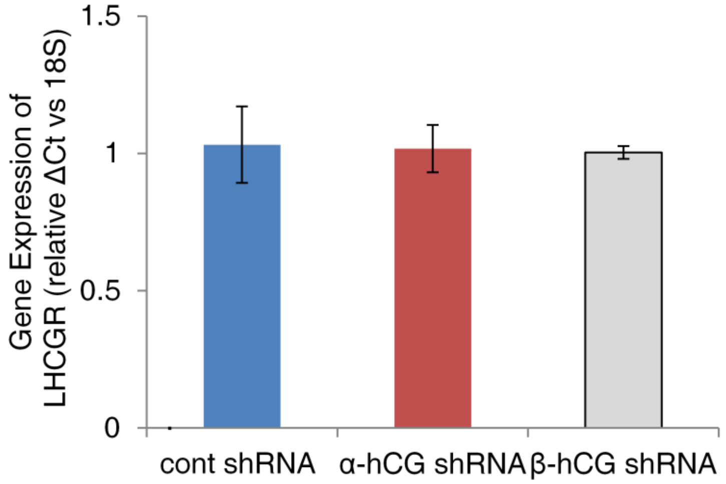

Gene Expression of LHCGR (relative ΔCt vs 18S)
1.5
1
0.5
0
cont shRNA
α-hCG shRNA
β-hCG shRNA

Figure S8

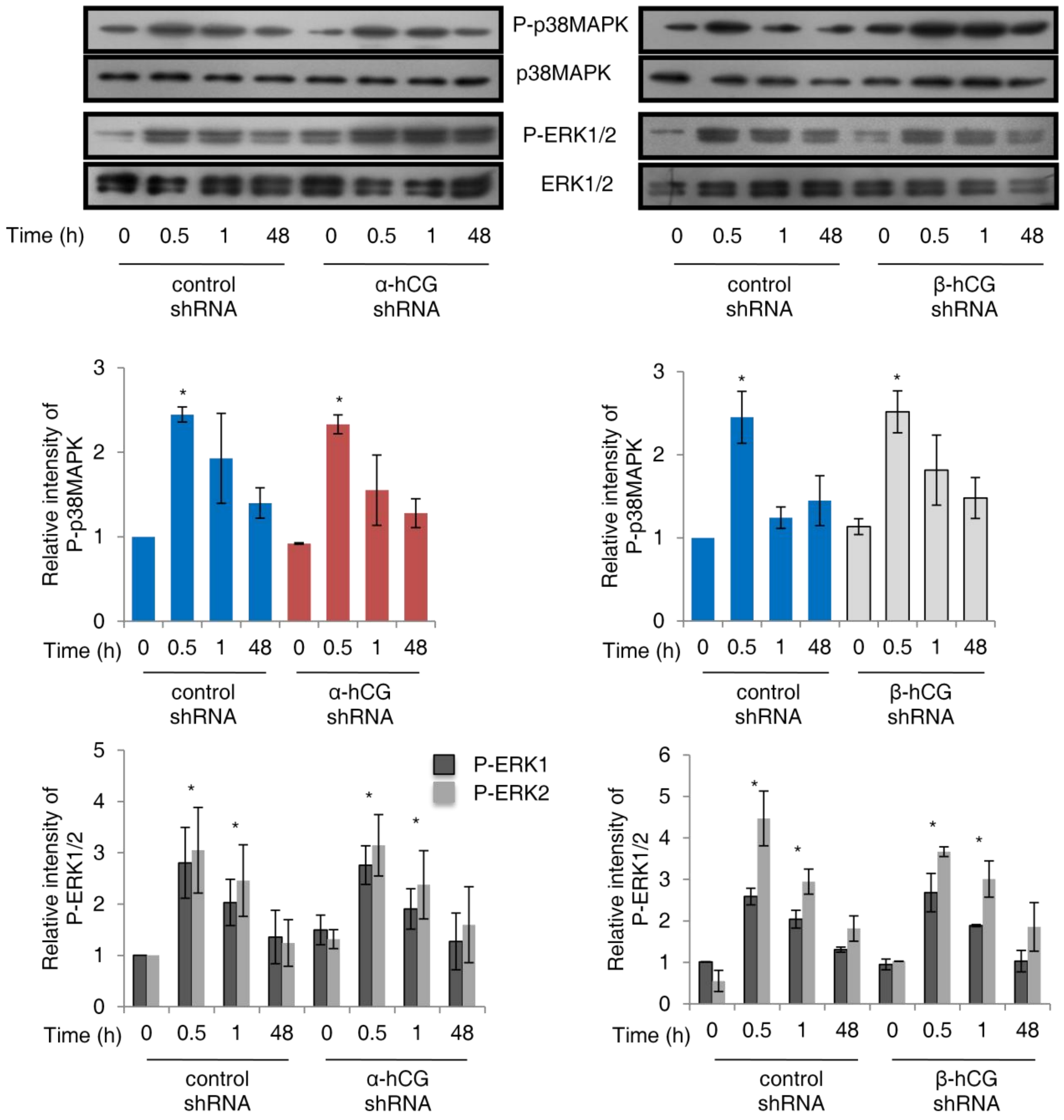

P-p38MAPK
p38MAPK
P-ERK1/2
ERK1/2
Time (h) 0 0.5 1 48 0 0.5 1 48
control shRNA
α-hCG shRNA
β-hCG shRNA
Relative intensity of P-p38MAPK
Relative intensity of P-ERK1/2
P-ERK1
P-ERK2

Figure S9

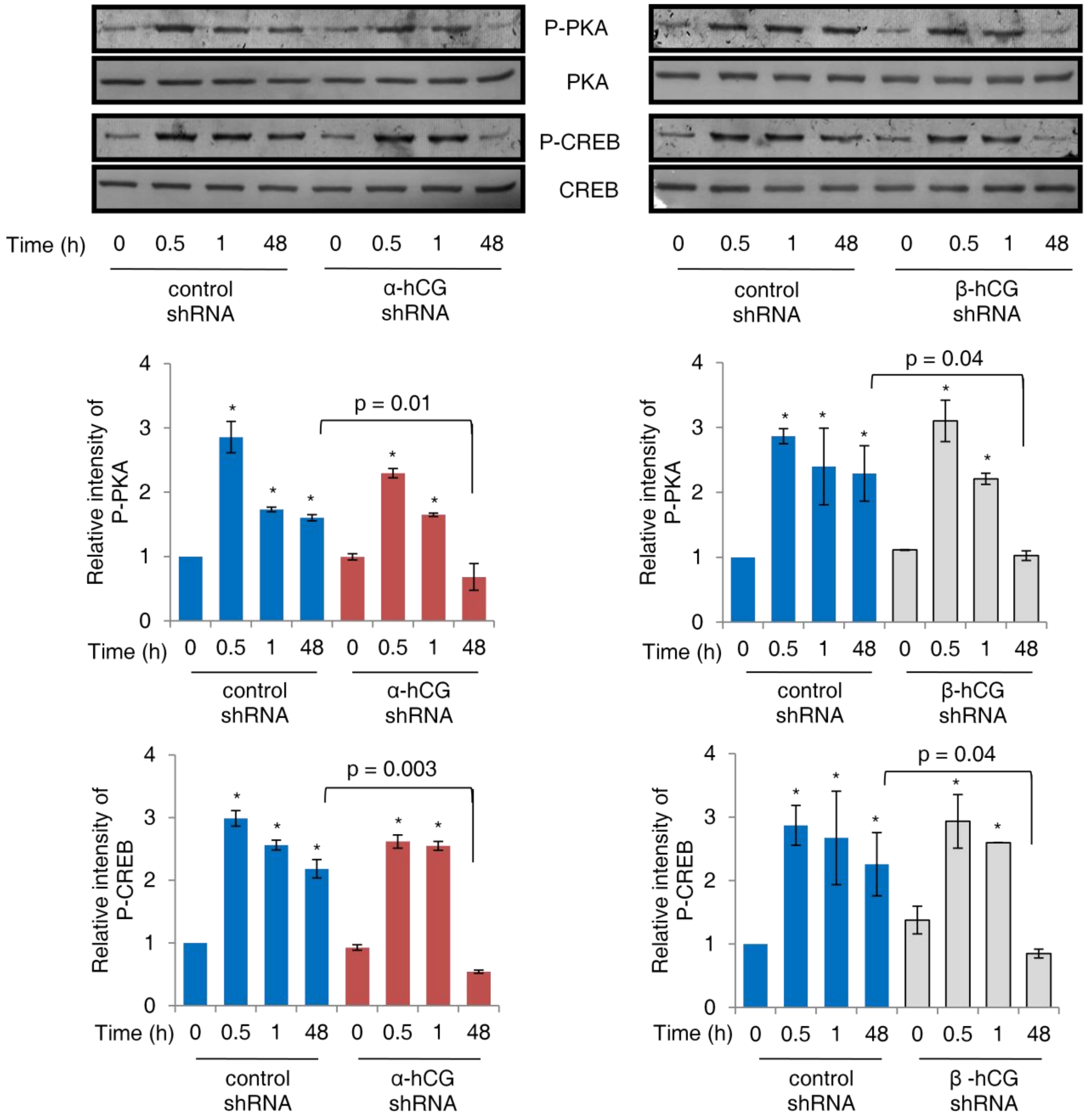
P-PKA
PKA
P-CREB
CREB
Time (h)
0
0.5
1
48
control shRNA
α-hCG shRNA
β-hCG shRNA
Relative intensity of P-PKA
Relative intensity of P-CREB
p = 0.01
p = 0.04
p = 0.003
p = 0.04
β -hCG shRNA

Figure S10

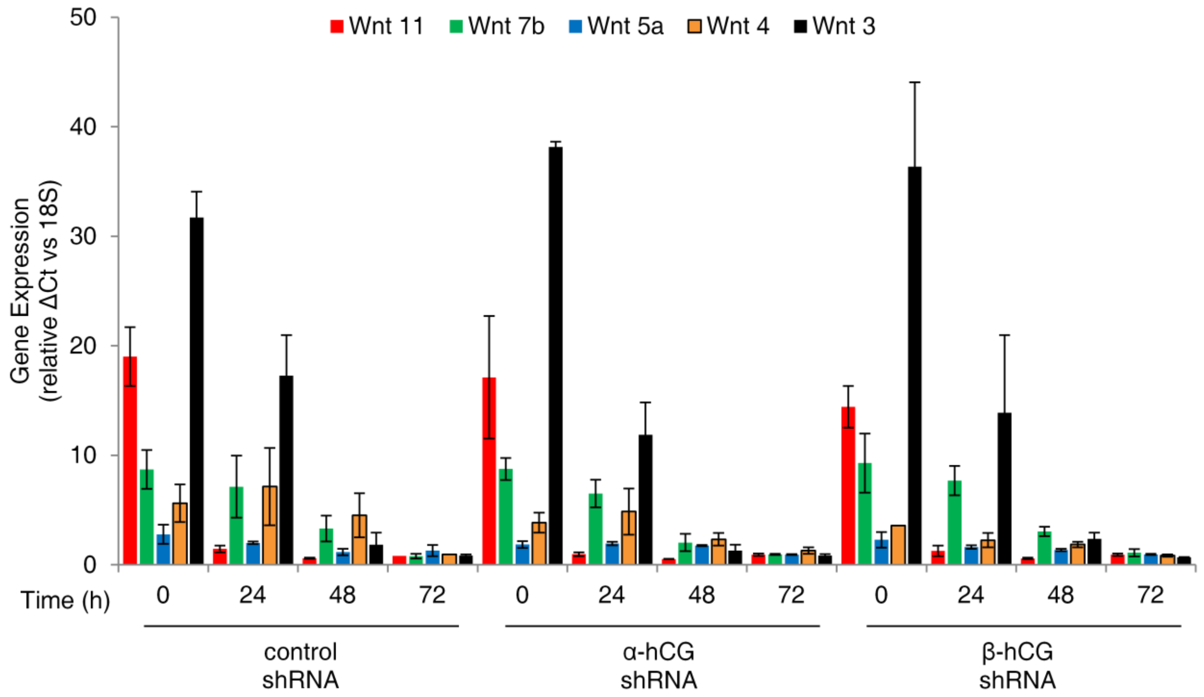

Figure S11

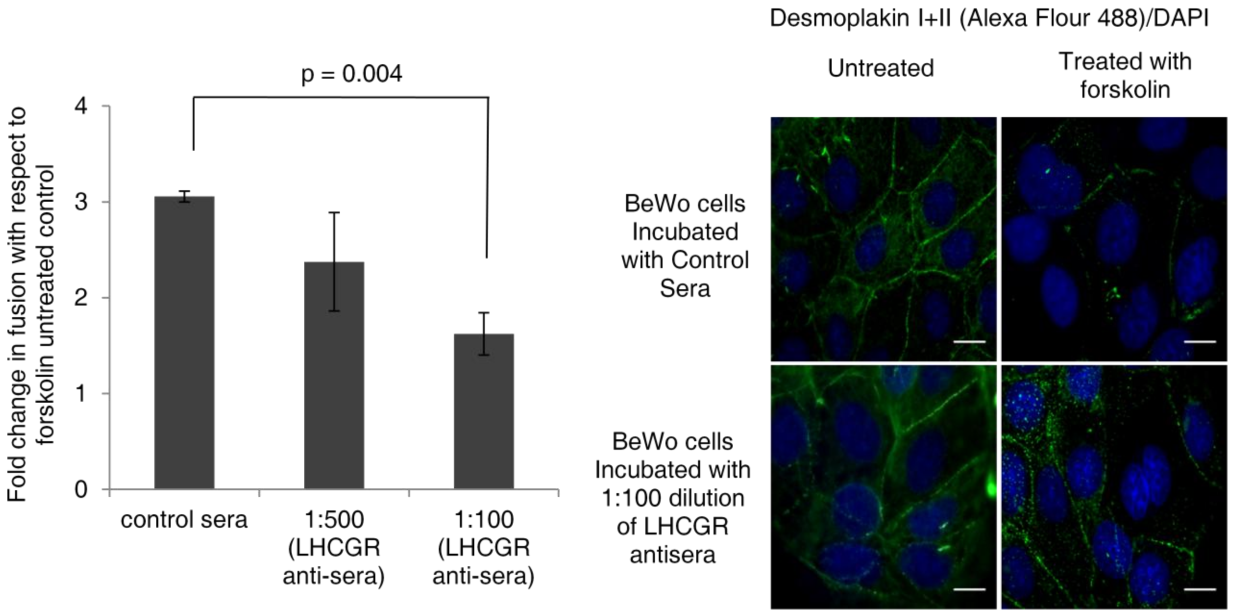

Figure S12

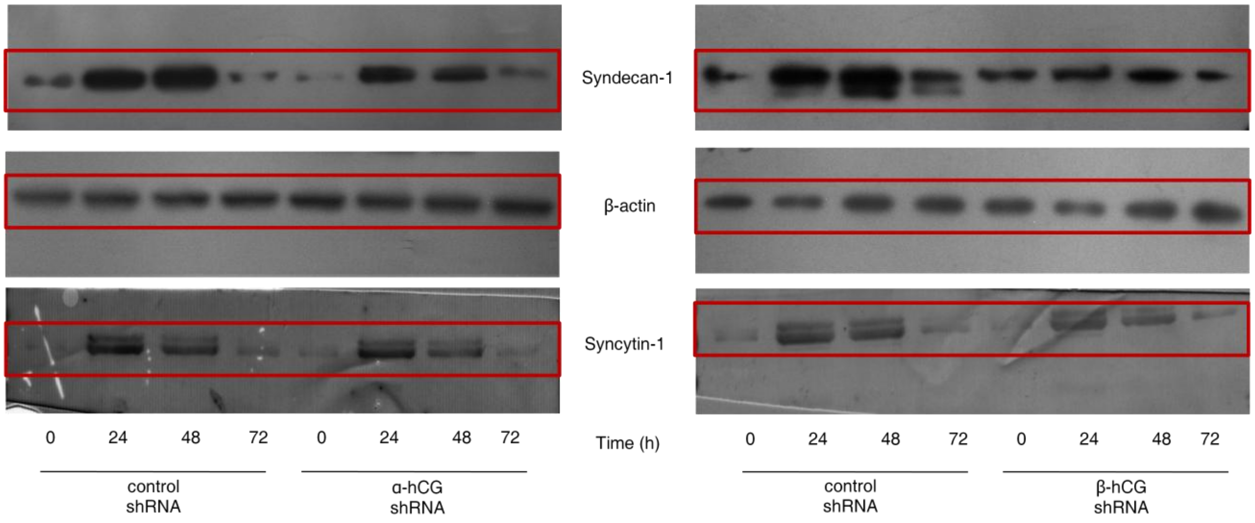

Figure S13

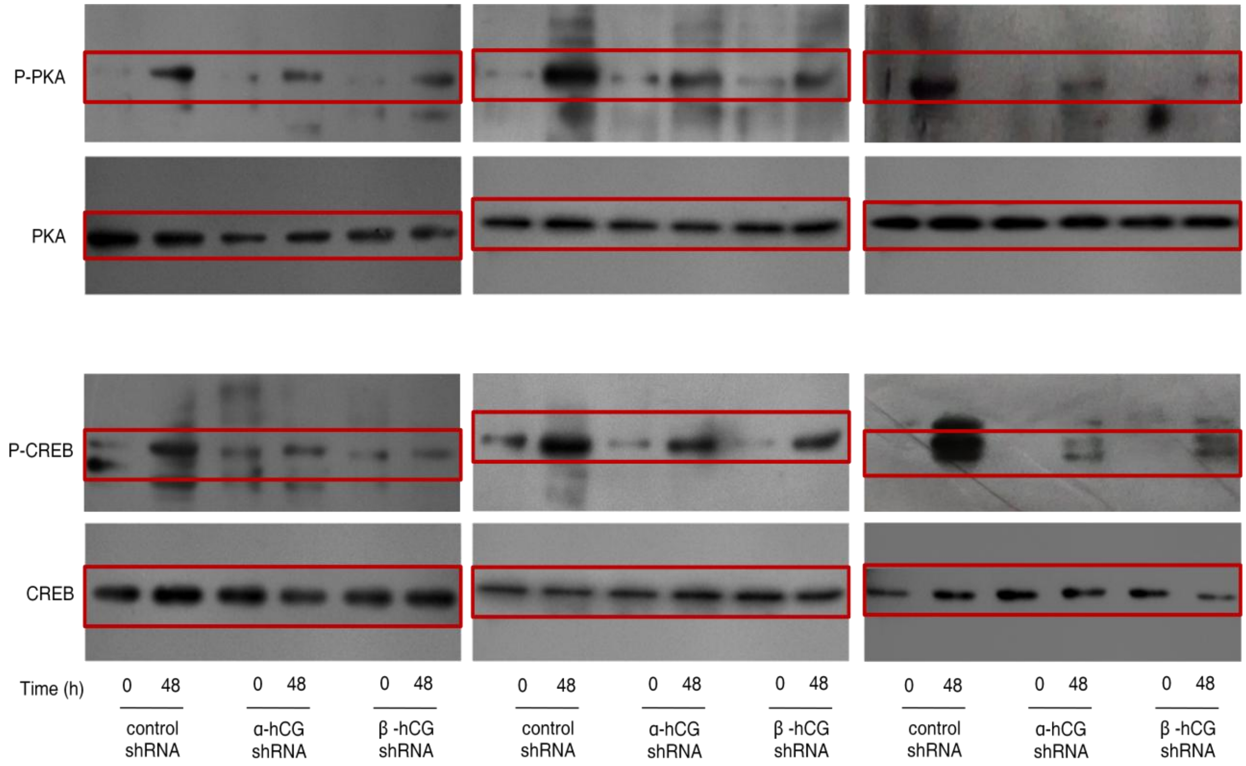

Figure S14

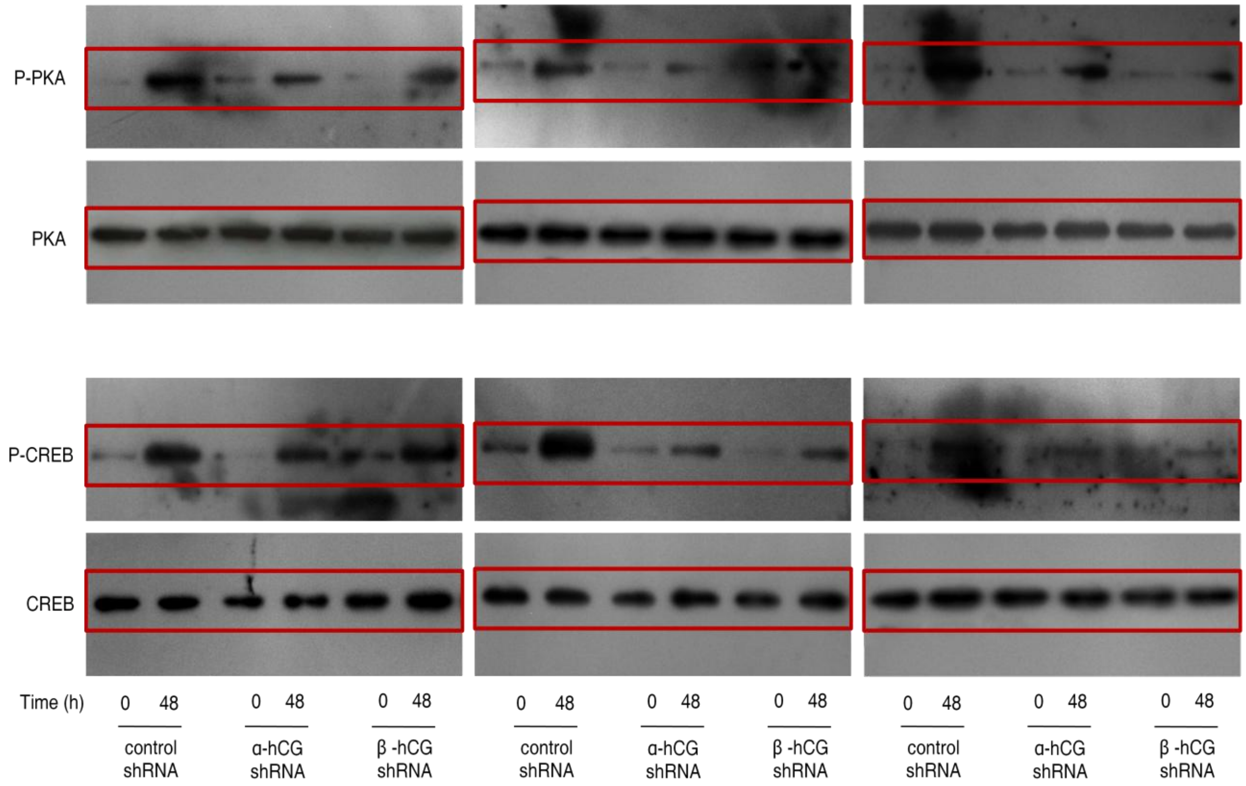
P-PKA
PKA
P-CREB
CREB
Time (h)
0 48
0 48
0 48
0 48
0 48
0 48
0 48
0 48
0 48
control shRNA
α-hCG shRNA
β -hCG shRNA
control shRNA
α-hCG shRNA
β -hCG shRNA
control shRNA
α-hCG shRNA
β -hCG shRNA

Figure S15

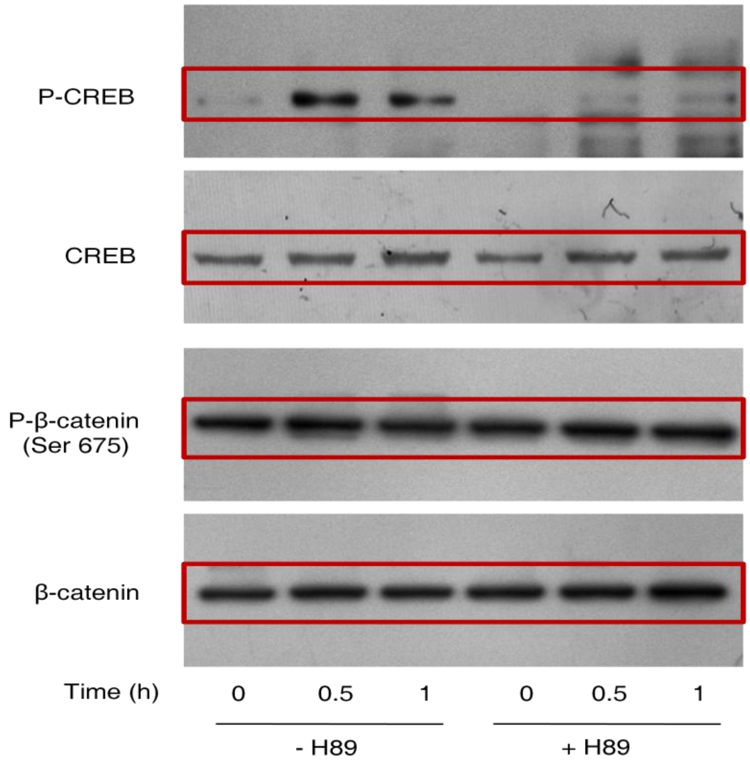
P-CREB
CREB
P-β-catenin (Ser 675)
β-catenin
Time (h)
0
0.5
1
0
0.5
1
- H89
+ H89

Table S1. Primer sequences used in quantitative real time PCR

Gene	Primer	Annealing temperature	Product size (bp)
hCG α	F 5′ ACCGCCCTGAACACATCCTGC 3′<br>R 5′ GCGTGCATTCTGGGCAATCCTGC 3′	60 °C	151
hCG β	F 5′ CCCCTTGACCTGTGATGACC 3′<br>R 5′ TATTGTGGGAGGATCGGGGT 3′	60 °C	120
Syndecan-1	F 5′ GAAAGCGACGTTCGGCCAC 3′<br>R 5′ CCGCTCTCTACTGCCGGATT 3′	60 °C	111
Syncytin-1	F 5′ GGTACATGGCACCTCTAGCCCCTAC 3′<br>R 5′ CAGGGATTGAAACATATGGCCTGAAGTTG 3′	60 °C	191
Wnt 3	F 5′ TGAACAAGCACAACAACGAG 3′<br>R 5′ CAGTGGCATTTTTCCTTCC 3′	60 °C	439
Wnt 4	F 5′ TACCTGGCCCAAGCTGTCGTC 3′<br>R 5′ CCCGAGTCCCTTGCGTCAC 3′	60 °C	244
Wnt 5a	F 5′ GGGAGGTTGGCTTGAACATA 3′<br>R 5′ GAATGGCACGCAATTACCTT 3′	58 °C	141
Wnt 7b	F 5′ GGGCCACCTGCTGAAGGAGAA 3′<br>R 5′ TTGACGAAGCAGCACCAGTGGAA 3′	60 °C	333
Wnt 10b	F 5′ GGGGTGGCTGTAACCATGAC 3′<br>R 5′ TTTTCAGTTACCACCTGGCG 3′	60 °C	139
Wnt 11	F 5′ GTAAGTGCCATGGGGTGTCT 3′<br>R 5′ GCTTCCAAGTGAAGGCAAAG 3′	60 °C	594
hPL	F 5′ CAGAAACAGGTGGGGTCAAG 3′<br>R 5′ CCACTAGGTGAGCTGTCCAC 3′	60 °C	133
CYP19A1	F 5′ GACGTCGCGACTCTAAATTGC 3′<br>R 5′ TGGGAGATGAGGGGTCCAAT 3′	60 °C	231
LHCGR	F 5′ AAAAACTGAGGAGGTATTTTGCAT 3′<br>R 5′ ATGCACTGAGACAGGGTTCC 3′	60 °C	486
18S	F 5′ GGAGAGGGAGCCTGAGAAAC 3′<br>R 5′ CCTCCAATGGATCCTCGTTA 3′	60 °C	171

Supporting Information

Supplementary Methods Information

Western blotting. It was essentially performed as described in *Methods* section of the main manuscript.

Desmoplakin I+II immunofluorescent staining. Cells (0.3×10^5/well) were grown on cover slips in 24-well culture plates in Ham's F-12 medium supplemented with 10% FBS. After 24 h, cells were washed and starved for 4 h with Ham's F-12 medium without FBS. Cells were treated with 25 µM forskolin (Sigma-Aldrich Inc., St. Louis, MO, USA) in medium supplemented with 1×ITS+1 (Sigma-Aldrich Inc.) for 0, 48 and 72 h, keeping appropriate vehicle control. After each time point, the cells were fixed with chilled methanol for 5 min at 4 °C and processed for desmoplakin I + II staining as described previously (Prakash, G.J., Suman, P., & Gupta, S.K. Relevance of syndecan-1 in the trophoblastic BeWo cell syncytialization. *Am J Reprod Immunol.* **66,** 385-393 (2011). Cover slips were mounted on glass slides using VECTASHIELD mounting medium H-1500 containing DAPI (Vector laboratories Inc., Burlingame, CA, USA) and subsequently screened for immunofluorescence under a fluorescent microscope (Nikon Instruments Inc., Melville, NY, USA) and images were captured using the Image Proplus software (Nikon).

Inhibition of LHCGR by rabbit polyclonal antibodies

LRR1-6 antiserum raised in rabbit against leucine rich repeats 1-6 of the human LHCGR (provided generously by Prof. Rajan Dighe, Indian Institute of Science, Bangalore, India) competitively inhibits hCG binding to human LHCGR. BeWo cells (0.3×10^5/well) were seeded

on cover slips in 24-well culture plates in Ham's F-12 medium supplemented with 10% FBS overnight. The cells were starved of FBS for at least 4 h before pre-treatment with LRR1-6 antiserum (1:100 and 1:500 dilutions) for 1 h in Ham's F-12 medium supplemented with 1× ITS+1 (Sigma-Aldrich Inc.). Normal rabbit serum at similar dilutions was used as an internal control. Subsequently, forskolin (25 µM; Sigma-Aldrich Inc.) was added to the medium, cells processed for desmoplakin I+II staining after 48 h and analyzed for fusion as described in the *Methods*.

Immunolocalization of syndecan-1/syncytin-1. Cells (0.3×10^5/well) were cultured over cover slips in 24-well culture plates, treated with forskolin (25 µM; Sigma-Aldrich Inc.) for 0, 48 and 72 h and further fixed with chilled methanol. For detection of syndecan-1, rabbit polyclonal antibody (Invitrogen Corp., Carlsbad, CA, USA) and for syncytin-1, goat polyclonal antibody (Thermo Fisher Scientific, Waltham, MA, USA) were used at a dilution of 1:100 in PBS containing 0.1% BSA. Subsequently, in case of syndecan-1 Alexa Fluor 488-labeled goat anti-rabbit IgG (Invitrogen Corp.) and for syncytin-1 Rhodamine-labeled donkey anti-goat IgG (Santa Cruz Biotechnology Inc., Dallas, Texas, USA) at an optimized dilution of 1:300 in PBS containing 0.1% BSA were used and cells were processed for examination of the fluorescence profile by mounting on glass slides using VECTASHIELD mounting medium H-1500 containing DAPI (Vector laboratories Inc.).

Cell viability and proliferation assay

To assess the effect of scrambled or α-/β-hCG shRNA treatment on cell viability and proliferation as compared to naïve BeWo cells, Sytox Red staining and [^{3}H]-thymidine

incorporation assays were performed respectively. For cell death determination, cells (0.1 × 10^6/well) were seeded in 6-well culture plates in Ham's F-12 medium with 10% FBS and cultured for 24 h. After which, they were trypsinized and processed for flow cytometry with Sytox Red (Invitrogen Corp.) staining. Live cells were Sytox Red negative and results were described as fold change in percent live cells with respect to naïve BeWo cells. Proliferation study was carried out by seeding cells (0.15 × 10^5/well) in a 96-well culture plate in Ham's F-12 medium with 10% FBS and were allowed to adhere overnight. Subsequently, [^{3}H]-thymidine (0.5 µCi/well; Perkin Elmer, Waltham, MA, USA) was added and cells were further cultured for 18 h at 37 °C in humidified 5% CO_2 incubator. After incubation, cells were processed, DNA harvested onto glass fiber filtermat and counted by means of scintillation spectroscopy (Betaplate, LKB-Pharmacia, Uppsala, Sweden). Results were expressed as fold change in proliferation with respect to naïve BeWo cells.

Quantitative reverse transcription-polymerase chain reaction. It was essentially performed as described in *Methods* section of the main manuscript.

Supplementary Figure Legends

Figure S1 Effect of either α- or β-hCG silencing in BeWo cells on the α- and β- subunits of hCG at protein level in response to forskolin. BeWo cells knockdown for α- and β-hCG were made using shRNA as described in *Methods*. To confirm target specific silencing at protein level, Western blotting was performed at 0, 48 and 72 h after forskolin treatment in control shRNA, α- and β-hCG shRNA treated cells with monoclonal antibodies against α- and β-hCG. Graphs represent the densitometric plots for α- and β-hCG proteins among control and silenced groups.

Band intensities were normalized with respect to β-actin and the data is expressed as fold change with respect to 0 h of control cells. The data is shown as mean ± s.e.m. of at least three experiments. Representative blots for the same are also shown. *$p < 0.05$ with respect to 0 h

Figure S2 Desmoplakin I+II immunofluorescent profile of control shRNA, α- and β-hCG shRNA treated cells on treatment with forskolin. Control shRNA, α- and β-hCG silenced cells were treated with 25 µM of foskolin and appropriate vehicle control for 0, 48 and 72 h and percent fusion was estimated by desmoplakin I+II staining. This figure gives a representative picture of desmoplakin I+II profile (green) using Alexa Fluor 488 conjugated secondary antibody and nuclear staining by DAPI (blue) at different time intervals for control, α- and β-hCG silenced cells with or without treatment with forskolin. Scale bar represents 20 µm.

Figure S3 Immunofluorescent profile of syndecan-1 and syncytin-1 in control shRNA, α- and β-hCG silenced BeWo cells on forskolin treatment. Control shRNA, α- and β-hCG silenced cells were treated for 0, 48 and 72 h with 25 µM forskolin and syndecan-1 (green) and syncytin-1 (red) were localized by immunofluorescence using Alexa Flour 488 and Rhodamine conjugated secondary antibodies respectively and nuclear staining by DAPI (blue). Images were compiled using Image Proplus software. Their fluorescence intensity was calculated using ImageJ software and represented as relative intensity with respect to 0 h control. Scale bar represents 20 µm. *$p < 0.05$ with respect to 0 h

Figure S4 Profile of hPL and CYP19A1 transcripts as fusion markers by qRT-PCR in control shRNA, α- and β-hCG silenced BeWo cells treated with forskolin**.** Control shRNA, α- and β-

hCG silenced cells were treated with forskolin (25 μM) for 0 and 72 h and its effect on the transcript level of hPL and CYP19A1 was analyzed by qRT-PCR as decribed in *Methods*. Each bar represents relative ΔCt values after normalization with the 18S rRNA, expressed as mean ± s.e.m. of three independent experiments performed in triplicates. *p < 0.05 with respect to 0 h

Figure S5 Effect of exogenous hCG treatment on the expression of various fusion associated proteins in naïve BeWo cells. BeWo cells were treated with hCG (5 IU/mL) for 24, 48 and 72 h. Cell lysates were prepared and profiles of syncytin-1 and syndecan-1 were determined by Western blotting. Subsequently, total RNA was also isolated and transcript levels of hPL and CYP19A1 were determined by qRT-PCR. In panel (a) graph represents expression profile of syndecan-1 and syncytin-1 on hCG treatment with β-actin used as an internal control. Values are expressed as mean ± s.e.m. of band intensities of three independent experiments. Representative blots for the same are also shown. Panel (b) represents qRT-PCR data showing transcript levels of hPL and CYP19A1 after 24, 48 and 72 h of hCG treatment. Each bar represents relative ΔCt values after normalization with the 18S rRNA, expressed as mean ± s.e.m. of three independent experiments performed in triplicates.*p < 0.05 with respect to 0 h

Figure S6 Cell viability and proliferation rate of α- and β-hCG silenced and control shRNA treated cells as compared with naïve BeWo cells. Naïve BeWo cells, α- and β-hCG silenced and control shRNA treated BeWo cells were cultured for 24 h and subsequently assessed for cell viability and proliferation. Cell death was assessed by flow cytometry using Sytox Red staining and cell proliferation was determined by incorporation of [^{3}H]-thymidine as described in supplementary methods. In panel (a) graph represents fold change in percent live cells (control

shRNA, α- and β-hCG silenced cells) with respect to naïve BeWo cells. The data is also expressed as contour diagram where Sytox Red negative are live cells. In panel (b) graph shows fold change in proliferation of control shRNA, α- and β-hCG silenced cells as compared to naïve BeWo cells.

Figure S7 LHCGR transcript level in the α- and β-hCG silenced and control shRNA treated cells. Total RNA was isolated from α- and β-hCG silenced and control shRNA treated cells and transcript level of LHCGR was determined by qRT-PCR. Graph represents qRT-PCR data showing transcript level of LHCGR in α- and β-hCG silenced and control shRNA treated cells. Each bar represents relative ΔCt values after normalization with the 18S rRNA, expressed as mean ± s.e.m. of three independent experiments performed in triplicates.

Figure S8 Profile of phosphorylated p38MAPK and ERK1/2 in α- and β-hCG silenced BeWo cells treated with forskolin. Control shRNA, α- and β-hCG silenced BeWo cells were treated with forskolin (25 µM) for varying periods of time (0, 0.5, 1 and 48 h) and Western blots were performed to study the activation of p38MAPK and ERK1/2 as described in *Methods.* Graphs represent the densitometric plots of P-p38MAPK (Thr180/Tyr182) and P-ERK1 (Thr202/Tyr204)/ P-ERK2 (Thr185/Tyr187). Band intensities were normalized with respect to their respective non-phosphorylated proteins and the data is expressed as fold change with respect to 0 h of control cells. The data is shown as mean ± s.e.m. of at least three experiments. Representative blots for the same are also shown. $^*p < 0.05$ with respect to 0 h

Figure S9 Expression profiles of activated PKA and CREB in control and α- and β-hCG silenced BeWo cells treated with forskolin. Control and α- and β-hCG silenced cells were treated with forskolin (25 μM) for varying period of time (0, 0.5, 1 and 48 h) and Western blotting for phophorylated and non-phosphorylated forms of PKA and CREB was performed as described in *Methods.* Graphs represent the densitometric plots of P-PKA (Thr197) and P-CREB (Ser133). Band intensities were normalized with respect to their respective non-phosphorylated proteins and the data is expressed as fold change with respect to 0 h of control cells. The data is shown as mean ± s.e.m. of at least three experiments. Representative blots for the same are also shown. $^*p < 0.05$ with respect to 0 h

Figure S10 Transcript levels of different Wnts during forskolin mediated differentiation of BeWo cell. Control shRNA, α- and β-hCG silenced cells were treated with forskolin (25 μM) for 24, 48 and 72 h. Subsequently, total RNA was prepared and qRT-PCR for various Wnt proteins was carried out. It represents the transcript profile for Wnt 3, 4, 5a, 7b and 11 at varying time points in the control and α- and β-hCG silenced BeWo cells. Each bar represents relative ΔCt values after normalization with the 18S rRNA, expressed as mean ± s.e.m. of three independent experiments performed in triplicates.

Figure S11 Effect of blocking LHCGR by rabbit polyclonal antibodies on forskolin-mediated BeWo cell fusion. BeWo cells were treated with LHCGR anti-serum at 1:100 and 1: 500 dilution for 1 h prior to forskolin (25 μM) treatment for 48 h using appropriate serum control as described in supplementary methods. Cell fusion was assessed by desmoplakin I+II staining and data is represented as fold change in fusion as compared with untreated control. Values are expressed as

mean ± s.e.m. of three independent experiments. Representative desmoplakin I + II staining profile has also been shown. Scale bar represents 20 μm.

Figure S12 Full length blots for Figure 2c

Figure S13 Full length blots for Figure 4

Figure S14 Full length blots for Figure 5

Figure S15 Full length blots for Figure 7b
